# Indapamide or chlorthalidone to reduce urine supersaturation for secondary prevention of kidney stones: protocol for a randomised, double-blind, cross-over trial (INDAPACHLOR)

**DOI:** 10.1136/bmjopen-2025-101594

**Published:** 2025-06-16

**Authors:** Martin Scoglio, Matteo Bargagli, Felix Rintelen, Marie Roumet, Sven Trelle, Daniel G Fuster

**Affiliations:** 1 Department of Nephrology and Hypertension, University of Bern, Bern, Switzerland; 2 Graduate School for Health Sciences, University of Bern, Bern, Switzerland; 3 Department of Clinical Research, University of Bern, Bern, Switzerland

**Keywords:** NEPHROLOGY, UROLOGY, Urolithiasis, Kidney & urinary tract disorders, Adult nephrology

## Abstract

**Introduction:**

Kidney stones constitute a major global healthcare problem and are characterised by high recurrence rates. Thiazide and thiazide-like diuretics (thiazides) have been the standard medical treatment for the prevention of kidney stone recurrence. This clinical routine has recently been challenged by the findings of the large NOSTONE trial that failed to show superiority of hydrochlorothiazide at doses up to 50 mg daily over placebo in preventing a composite of clinical or radiological recurrence in patients at high risk of kidney stone recurrence. If these results also apply to the longer-acting and more potent thiazides indapamide and chlorthalidone remains unknown. No head-to-head comparison of different thiazides for kidney stone recurrence prevention or for the established proxies of recurrence risk, urine relative supersaturation ratios, has ever been conducted.

**Methods and analysis:**

INDAPACHLOR is a single-centre, randomised, double-blind, cross-over trial evaluating the efficacy of indapamide or chlorthalidone compared with hydrochlorothiazide in lowering urine relative supersaturation ratios for calcium oxalate and calcium phosphate in individuals with idiopathic calcium kidney stones. Participants will be allocated to indapamide 2.5 mg once daily, chlorthalidone 25 mg once daily and hydrochlorothiazide 50 mg once daily in a random sequence. The three consecutive active treatment periods of 28 days each will be separated by wash-out periods of 28 days. Inclusion criteria are age ≥18 years and ≥2 stone episodes in the last 10 years with calcium-containing kidney stones (containing ≥50% of calcium oxalate, calcium phosphate or a mixture of both). Patients with secondary causes of calcium kidney stones are excluded. The primary outcomes are the changes in the relative supersaturation ratios of calcium oxalate and calcium phosphate from baseline to day 28 of each treatment period. Secondary outcomes include changes in 24 hours urine and blood parameters from baseline to day 28 of each treatment period. The study targets enrolment of 99 participants to achieve 80% power for detecting a 20% reduction in the relative supersaturation ratios of calcium oxalate and calcium phosphate when treated with indapamide or chlorthalidone and hydrochlorothiazide.

**Ethics and dissemination:**

The study was approved by the Ethics Commission Bern, Switzerland, and the Competent Authority Swissmedic. Results will be disseminated through peer-reviewed publications and conference presentations.

**Trial registration numbers:**

ClinicalTrials.gov (NCT06111885) and Swiss National Clinical Trials Portal (SNCTP000006156).

**Protocol version:**

Version 4.0, 29 November 2024.

**STRENGTHS AND LIMITATIONS OF THIS STUDY**

- Rigorous comparison of the three most frequently used thiazides for kidney stone prevention.
- Randomised, double-blind, cross-over design.
- Relative supersaturation ratios, validated proxies of stone recurrence risk, as primary outcome.
- Single-centre trial with insufficient sample size and follow-up time for the evaluation of clinical endpoints (stone events).

## Introduction

 Nephrolithiasis is the most common kidney disease worldwide, affecting up to 20% of men and 10% of women during their lifetime.^1^ Both the prevalence and incidence of kidney stones have risen in recent decades across various demographic groups, regardless of age, sex or ethnicity.^2 3^ Kidney stones are not only highly recurrent and extremely painful but also lead to significant costs, increased morbidity and reduced quality of life.^4^,^6^

Most kidney stones consist of calcium oxalate, calcium phosphate or a combination of both. The most common metabolic abnormality in patients with kidney stones is hypercalciuria.^7^ Supersaturation, the presence of a salt in solution at a concentration exceeding its own solubility, is the driving force for crystallisation and therefore kidney stone formation. At a supersaturation <1, crystals dissolve; at a supersaturation >1, crystals form.^8^ Relevant urine supersaturations for calcium-containing kidney stones include calcium oxalate and calcium phosphate. Urinary calcium excretion contributes to supersaturation of both calcium oxalate and calcium phosphate, thus increasing the risk of forming calcium oxalate and calcium phosphate stones.^9^ In clinical routine, supersaturations are approximated using a set of 14 parameters measured in 24-hour urines. The most common software employed for this purpose is EQUIL2, and the calculated supersaturations are referred to as relative supersaturation ratios.^10^ Urine supersaturations are highly correlated with kidney stone composition and well-established predictors of stone recurrence.^9 11^ Interventions that reduced stone events in randomised controlled trials and prospective studies closely correlated with reductions in urine relative supersaturation ratios.^12^

Thiazide and thiazide-like diuretics, commonly known as ‘thiazides’, reduce urine calcium and have been the cornerstone of pharmacologic prevention of kidney stone recurrence for more than 50 years.^13^,^15^ The efficacy of thiazides in the prevention of kidney stone recurrence has been evaluated in several randomised-controlled trials.^16^,^27^ Thiazides reduced kidney stone recurrence in most of these trials,^16^,^2325^ but all of these trials had major methodological limitations.^28^,^30^ To address these issues, NOSTONE, a double-blind, randomised, placebo-controlled trial, was recently conducted to assess the efficacy of incremental doses of hydrochlorothiazide in preventing recurrence in individuals with recurrent idiopathic calcium stones.^29 31 32^ Over a 3-year follow-up period, recurrence rates were similar among groups, with no clear dose-dependent relationship. Yet, adverse events (hypokalaemia, gout, new-onset diabetes, skin reactions and increased plasma creatinine) were more frequently reported in patients treated with hydrochlorothiazide. Overall, these findings suggest that hydrochlorothiazide, the most widely used and best studied thiazide, is not effectively preventing kidney stone recurrence and is associated with notable side effects.

The lack of efficacy of hydrochlorothiazide in NOSTONE, recent concerns about skin cancer associated with long-term use of hydrochlorothiazide^33^,^35^ and the absence of effective and well-tolerated alternatives for recurrence prevention will likely lead to a prescription shift to the two thiazide-like diuretics indapamide and chlorthalidone, which are more potent and have a significantly longer half-life compared with hydrochlorothiazide. Both indapamide (at a dose of 2.5 mg daily) and chlorthalidone (at doses of 25 mg or 50 mg daily) reduced kidney stone recurrence in one past randomised controlled trial each, but both trials had considerable methodological deficiencies, such as small size, open-label design, lack of intention-to-treat analysis, outdated dietary recommendations and low sensitivity and specificity imaging.^16 17^ Recurrence risk reduction seemed to be more pronounced compared with hydrochlorothiazide trials, but the large variation in trial design and methodological issues of all past thiazide trials preclude definitive conclusions. No head-to-head comparison of different thiazides for kidney stone recurrence prevention or for the established proxies of recurrence risk, urine relative supersaturation ratios of calcium oxalate and calcium phosphate, has ever been performed.

We conducted a systematic literature search on the impact of indapamide or chlorthalidone on urine calcium, urine citrate and urine relative supersaturation ratios of calcium oxalate and calcium phosphate (table 1).

**Table 1:** Systematic review of the impact of indapamide and chlorthalidone on urinary composition and supersaturations

Variable	Dosage (mg/day)	Comparison	Exposure	Mean absolute change	Mean percentage change	First author, year
Indapamide						
Relative supersaturation ratio of calcium oxalate	2.5	Baseline	36 months	−4.08	−54%	Borghi, 1993^16^
Relative supersaturation ratio of calcium phosphate	2.5	Baseline	36 months	−0.78	−61%	Borghi, 1993^16^
Urine calcium (mmol/24 hours)	1.5	Baseline	6–18 months	−4.90 to −4.55	−53% to −50%	Alonso, 2012^36^
	2.5	Baseline	3 months	−2.69	−44%	Martins, 1996^38^
	2.5	Baseline	6–36 months	−4.64 to −3.74	−48% to −39%	Borghi, 1993^16^
	2.5	Baseline and placebo	7 days	−2.70 to −1.80	−44% to −35%	Borghi, 1988^37^
	2.5	Baseline	3 months	−6.86 to −6.46	−56% to −52%	Lemieux, 1986^39^
Urine citrate (mmol/24 hours)	1.5 mg	Baseline	6–18 months	−1.31 to −0.81	−32% to −20%	Alonso, 2012^36^
	2.5 mg	Baseline	six to 36 months	−0.10 to +0.04	−3.9% to +1.5%	Borghi, 1993^16^
	2.5	Baseline	3 months	−0.01	−1%	Martins, 1996^38^
Chlorthalidone						
Relative supersaturation ratio of calcium oxalate	Not available	Not available	Not available	Not available	Not available	Not available
Relative supersaturation ratio of calcium phosphate	Not available	Not available	Not available	Not available	Not available	Not available
Urine calcium (mmol/24 hours)	25	Baseline	6 months	−4.62	−42%	Wolfgram, 2013^40^
	50–100	Baseline	3 months	−3.99 to −1.95	−56% to −29%	Coe, 1988^41^
	25–50	Baseline	36 months	−1.87 to −1.65	−28% to −22%	Ettinger, 1988^17^
	/	Baseline	34 months	/	−36%	Lockefeer, 1977^42^
Urine citrate (mmol/24 hours)	25	Baseline	6 months	−0.92	−19%	Wolfgram, 2013^40^

Articles were searched from accessible online databases (PubMed, the Cochrane Library and Web of Science). The articles of interest were obtained using the following search terms: (“indapamide” OR “chlorthalidone” OR “thiazide*” OR “thiazide-like diuretics”) AND (“citrate” OR “calcium” OR “calciuria” OR “hypercalciuria” OR “supersaturation*” OR “RSR” OR “RSS” OR “SS”). Only articles written in English language and with available full text were included.

Indapamide at daily doses of 1.5–2.5 mg resulted in 20–56% reductions of urine calcium compared with baseline without a clear dose-response effect.^1636^,^39^ For chlorthalidone at daily doses of 25–100 mg, reductions of urine calcium of 22–56% compared with baseline have been reported again, without a clear dose–response effect.^1740^,^42^ These values exceed urine calcium reductions observed with hydrochlorothiazide in NOSTONE (9–17% compared with baseline, 15–16% compared with placebo).^31 32^ With respect to urine citrate, no changes and up to a reduction of 32% compared with baseline have been reported with indapamide without a clear dose–response effect. With 25 mg chlorthalidone daily, a reduction of urine citrate of 19% compared with baseline has been reported in a single study. In patients receiving high doses of hydrochlorothiazide (25 mg or 50 mg once daily), urine citrate tended to be lower compared with baseline and to patients receiving placebo, but differences were not significant.^29^ We found no data on the effects of chlorthalidone on relative supersaturation ratios in humans. For indapamide, a daily dose of 2.5 mg induced a 54% reduction in relative supersaturation ratio of calcium oxalate and a 22% reduction in relative supersaturation ratio of calcium phosphate compared with baseline. In NOSTONE patients receiving hydrochlorothiazide, there were no significant reductions in relative supersaturation ratios of calcium oxalate and calcium phosphate compared with baseline and patients receiving placebo. Thus, indapamide and chlorthalidone may be more effective in reducing urine calcium than hydrochlorothiazide. However, due to the large variability of urine citrate data reported, the paucity of supersaturation data and the lack of direct comparisons, it remains unclear if indapamide and chlorthalidone are superior to hydrochlorothiazide in reducing relative supersaturation ratios of calcium oxalate and calcium phosphate. This critical knowledge gap creates the need for a randomised trial that directly compares the efficacy of different thiazides in reducing the relevant relative supersaturation ratios of calcium oxalate and calcium phosphate in individuals with idiopathic calcium kidney stones.

## Methods and analysis

### Research hypothesis

**Figure 1:**
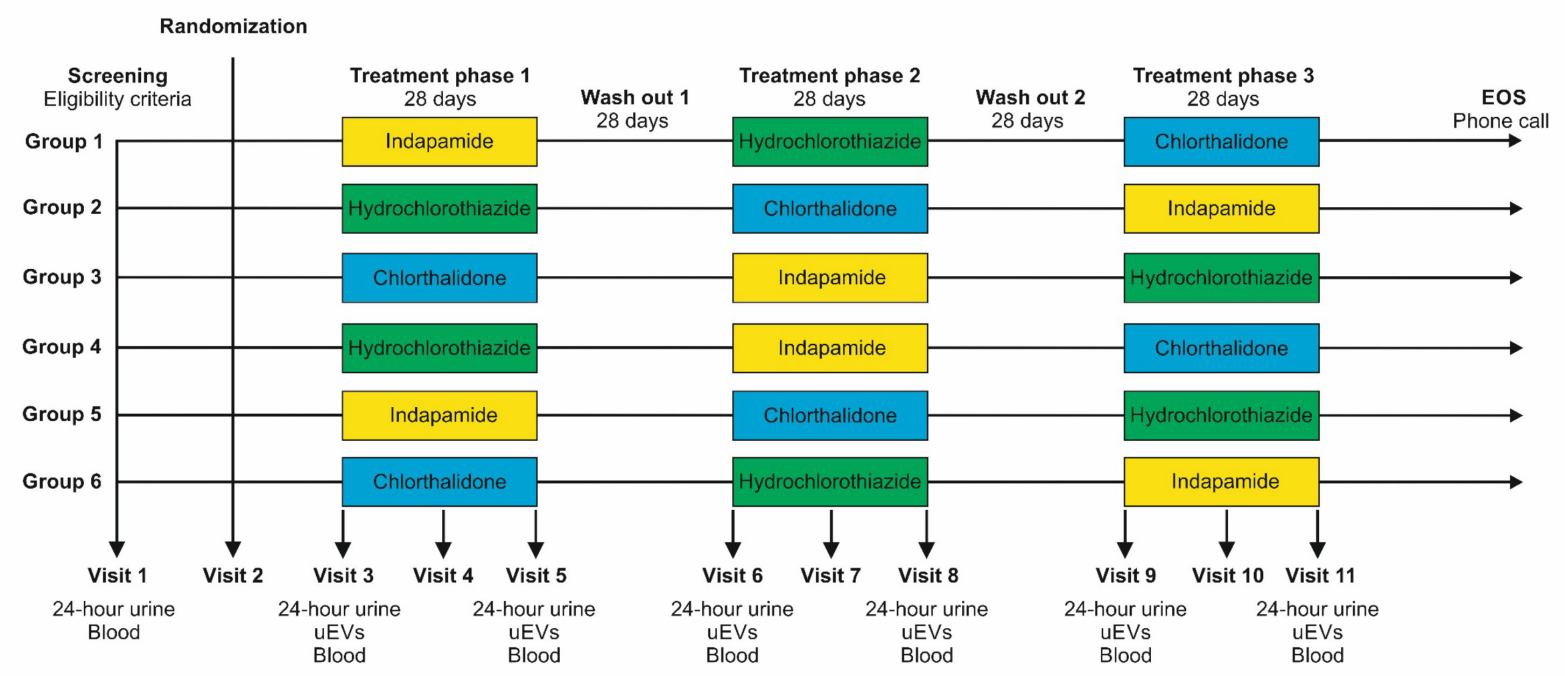
Flow chart of the study design. Patients will be randomised to one of six treatment sequences with indapamide 2.5 mg, chlorthalidone 25 mg or hydrochlorothiazide 50 mg once daily. Note that treatment periods and wash-out phases may be prolonged, if needed. At visits 4, 7 and 10, plasma potassium will be measured, and if <3.5 mmol/L, a substitution with KCl will be initiated. EOS, end of study; uEVs, urinary extracellular vesicles.

We hypothesise that at least one of the two thiazide-like diuretics indapamide and chlorthalidone has better prophylactic potential than hydrochlorothiazide in patients with recurrent calcium kidney stones. To test this hypothesis, we will conduct a randomised, double-blind, three-treatment, three-period, six-sequence cross-over trial (figure 1).

### Study objectives

The purpose of this study is to assess if indapamide or chlorthalidone is a promising candidate compound for reducing the risk of kidney stone recurrence to decide whether one (or both) is worthwhile to be evaluated in a large randomised-controlled trial with long-term follow-up and clinically relevant endpoints.

#### Primary objective

The primary objective is to determine if indapamide or chlorthalidone is superior to hydrochlorothiazide in reducing urine relative supersaturation ratios of calcium oxalate or calcium phosphate at 28 days as an indicator of the prophylactic potential.

#### Secondary objectives

To assess the impact of indapamide 2.5 mg daily, chlorthalidone 25 mg daily or hydrochlorothiazide 50 mg daily on supplementary urine and blood parameters including those to assess selected safety aspects at 28 days.

#### Safety objectives

Description of the safety of the interventions in the context of this study (taking into account that sample size and study duration do not allow for a conclusive safety profiling).

#### Exploratory objectives

To obtain a mechanistic understanding of potential effects observed with the three thiazides by analysing the abundance of the NaCl cotransporter in urinary extracellular vesicles at 28 days. Furthermore, we aim to set up a biobank for analyses of additional parameters that might be considered of relevance in the future.

### Study design

The INDAPACHLOR trial is a randomised, double-blind, monocentric, cross-over, exploratory study designed to compare the effects of indapamide, chlorthalidone and hydrochlorothiazide on urinary relative supersaturation ratios in individuals with recurrent idiopathic calcium kidney stones. Participants will be randomised in a 1:1:1 ratio to receive each of the three study drugs—indapamide (2.5 mg daily), chlorthalidone (25 mg daily) and hydrochlorothiazide (50 mg daily)—in a random sequence (figure 1). Randomisation lists will be prepared by an independent statistician at the Department of Clinical Research of the University of Bern to ensure allocation concealment. Participants, investigators including all staff interacting with participants, and statisticians will remain blinded to treatment allocation until all analyses are complete. Each treatment phase will last 28 days and will be followed by a washout period of 28–56 days to eliminate any carryover effects (plasma half-life of chlorthalidone: 40–50 hours; hydrochlorothiazide: 6–15 hours; indapamide: 14–18 hour). All drugs will be administered orally once daily.

### Study outcomes

#### Primary outcome

The primary objective will be addressed by evaluating two primary outcomes. Both outcomes will be assessed separately as they reflect different mechanisms and are both of relevance to assess the prophylactic potential:

- Change in relative supersaturation ratio of calcium oxalate from baseline to day 28 of each treatment period.
- Change in relative supersaturation ratio of calcium phosphate from baseline to day 28 of each treatment period.

Relative supersaturation ratios will be calculated by the EQUIL2 program on measurement of the relevant parameters in 24-hour urines as this calculation method was shown to be the best predictor of kidney stone recurrence.^10 43^

#### Secondary outcomes

Change from baseline to day 28 of each treatment period of the following parameters:

- Blood: Sodium, potassium, chloride, total and ionised calcium, magnesium, phosphate, creatinine, urea, uric acid, venous blood gas analysis, parathyroid hormone, 25-OH vitamin D, 1,25-OH vitamin D, glucose, haemoglobin A1c.
- 24-hour urine: Sodium, potassium, chloride, calcium, magnesium, phosphate, creatinine, urea, uric acid, oxalate, citrate, sulfate, ammonium, bicarbonate, pCO_2_ (partial pressure of carbon dioxide), pH, volume.

#### Other outcomes of interest

- Exploratory outcomes: Change in the abundance of total and phosphorylated NaCl cotransporters in urinary extracellular vesicles from baseline to day 28 of each treatment period.

#### Safety outcomes

Safety will be described using the following parameters:

- Serious adverse events: We will collect, fully investigate and document all serious adverse events in the source documents and the electronic case report forms for all participants from the date of signature of the informed consent form until the last protocol-specific procedure has been completed, including a safety follow-up period of 30 days after the last treatment period. The definition of what constitutes a serious adverse event follows standard definitions of the International Council for Harmonisation of Technical Requirements for Pharmaceuticals for Human Use (ICH) guidelines.^44^
- Prespecified adverse events of special interest: Adverse events of special interest are prespecified adverse events that may occur during investigational medical product intake and may or may not be related to the treatment. The following events are considered as adverse events of special interest: Hypokalaemia (defined as plasma potassium <3.5 mmol/L), gout or plasma uric acid levels requiring uric acid lowering therapy, and new onset diabetes mellitus (defined as haemoglobin A1c ≥6.5% or initiation of antidiabetic medication).
- Adverse events that require discontinuation of treatment: Use of prohibited medication indicated during the study (table 2), severe hypokalaemia (plasma potassium <2.5 mmol/L), uric acid lowering therapy, pregnancy.

**Table 2:** Eligibility criteria for the INDAPACHLOR trial

**Inclusion criteria**	
Patients fulfilling all the following inclusion criteria are eligible for the study:	
1	Written informed consent
2	Age 18 years or older
3	Recurrent kidney stone disease (2 or more stone episodes in the last 10 years prior to randomisation)
4	Past kidney stone containing 50% or more of calcium oxalate, calcium phosphate or a mixture of both
**Exclusion criteria**	
The presence of any one of the following exclusion criteria will lead to exclusion:	
1	Patients with secondary causes of recurrent calcium kidney stones including severe eating disorders (anorexia or bulimia), chronic bowel disease, intestinal or bariatric surgery, sarcoidosis, primary hyperparathyroidism, chronic urinary tract infection
2	Patients taking the following medications: Thiazide or loop diuretics, carbonic anhydrase inhibitors (including topiramate), xanthine oxidase inhibitors, alkali, active vitamin D (calcitriol or similar), calcium supplementation, bisphosphonates, denosumab, teriparatide, sodium-glucose cotransporter 2 (SGLT2) inhibitors, strong CYP3A4 inhibitors or inducers (may affect indapamide metabolism), lithium.
3	Patients with chronic kidney disease, defined as Chronic Kidney Disease Epidemiology Collaboration estimated glomerular filtration rate <30 mL/min
4	Patients with glomerulonephritis
5	Patients with the following biochemical imbalances: severe hypercalcaemia (> 2.8 mmol/L), therapy-resistant hypokalaemia or conditions with increased potassium loss, severe hyponatraemia (<130 mmol/L), symptomatic hyperuricaemia
6	Patients with hepatic encephalopathy or severe liver insufficiency
7	Patients with severe cardiac insufficiency
8	Patient with a recent cerebrovascular event
9	Patients with a solid organ transplant
10	Pregnant and lactating women (a urine pregnancy test must be performed for women of childbearing potential, defined as women who are not surgically sterilised/hysterectomised and/or who are postmenopausal for less than 12 months)
11	Previous (within 3 months prior to randomisation) or concomitant participation in another interventional clinical trial
12	Previous participation in the INDAPACHLOR trial
13	Inability to understand and follow the protocol
14	Allergy to any of the study drugs

- Vital signs: We will regularly assess vital signs (heart rate, systolic and diastolic blood pressure at the right arm after at least 5 min at rest) at all in-person visits (figure 1).

### Study site

The trial will be conducted at Inselspital, Bern University Hospital, Switzerland, which also functions as the sponsor of the trial. This is a tertiary care centre with around 900 beds and a catchment area of ~1.5 Mio. The Department of Nephrology and Hypertension at the Inselspital receives ~300 new referrals of patients with kidney stones per year and has ~1500 patients with recurrent kidney stones in annual follow-up.

### Study population

The study aims to recruit 99 adults aged 18 years or older who have experienced at least two episodes of calcium kidney stones in the last 10 years prior to randomisation. Eligibility criteria are depicted in table 2. If available medical history indicates eligibility for study participation, the individual will be informed in detail about the study by the responsible investigator. Inclusion will take place only on receipt of written informed consent (see documents ‘Participant consent form for the study’ and ‘Participant consent form for the biobank’ under online supplemental material) and complete fulfilment of all eligibility criteria. All study participants will receive a flat rate of 200 Swiss francs to compensate for travel costs associated with frequent study site visits. For patients assuming one of the medications outlined in table 2, this needs to be stopped, and a 28-day wash-out period is required prior to randomisation.

### Study assessments

The target population consists of individuals with a history of recurrent calcium-containing kidney stones. Recruitment will take place among outpatients, referred to our stone clinic for metabolic work-up or that are already in regular follow-up at our clinic. All blood analyses will be performed after at least 6 hours of fasting. Urine and blood analyses will be performed at the Central Laboratory of the Bern University Hospital using standard laboratory methods. Urine collections will be performed under paraffin oil with thymol as an additive. Urine pH will be measured by an electrode pH meter that will be calibrated daily.

Prior to randomisation, patients will undergo a screening visit to assess their eligibility for study participation, which includes an evaluation of their health status and a review of their stone history. Only stone composition analysis results based on the two gold standard methods, infrared spectroscopy or X-ray diffraction, will be accepted.^45^ During screening, a 24-hour urine and a fasting blood sample will be obtained in participants without nephrolithiasis work-up within the last 3 months. Patients will collect 24-hour urines at the beginning (the day before treatment start) and at the end of each treatment period (last day of intake) to measure urine parameters. Blood samples will be collected at the beginning (the first treatment day, before intake) and at the end of each treatment period (last day of intake). Systolic and diastolic blood pressure, heart rate, height (measured only once at the screening visit) and body weight will also be recorded at these time points. Fasting second morning urines will be collected at the beginning (the first treatment day, before intake, but after the end of the 24-hour urine collection) and at the end of each treatment period (on the last day of intake, after the end of the 24-hour urine collection) for isolation of urinary extracellular vesicles. At the beginning (the first treatment day, before intake) and at the end of each treatment period, blood and an aliquot of the 24-hour urine will be collected for the INDAPACHLOR biobank for future exploratory research projects. Urinary extracellular vesicles will be isolated in all participants at baseline and at the end of each treatment period from fasting second morning urines supplied with protease and phosphatase inhibitors, following established protocols.^46 47^ Total and phosphorylated NaCl cotransporters will be detected and quantified on immunoblots with highly sensitive and specific antibodies, normalised to the urinary extracellular vesicles housekeeping proteins Alix and CD9.^46^,^48^

### Investigational medicinal product (IMP)

Identical capsules containing 2.5 mg indapamide, 25 mg chlorthalidone or 50 mg hydrochlorothiazide will be supplied by Apotheke Dr. Hysek AG (Biel, Switzerland) according to applicable regulations. Capsules will be provided in bottles containing 35 capsules each and labelled with trial-specific labels according to ‘Manufacturing of IMP’ Volume IV of the EU guideline to Good Manufacturing Practice.^49^ Medication will be stored in a securely locked cabinet. Access will be limited to investigators and their designees. Capsules will be taken once daily per orally in the morning for 28 days. No dose adjustments are foreseen. Pill counts will be conducted at the end of each treatment period in all participants.

### Comedication and additional treatment

Hypokalaemia (defined as plasma potassium <3.5 mmol/L) is a very common side effect of thiazides and is causally linked to the development of hypocitraturia. Hence, we will ensure that all patients remain normokalaemic during the trial (plasma potassium 3.5–4.5 mmol/L). For this, a blood draw will be performed in the second week of each treatment period. In the case of mild hypokalaemia (3.0–3.4 mmol/L), supplementation with 40 mmol oral potassium chloride daily will be started. In the case of moderate hypokalaemia (2.5–2.9 mmol/L), supplementation with 80 mmol oral potassium chloride daily will be started. In case of severe hypokalaemia (plasma potassium <2.5 mmol/L), the patient will be withdrawn from the study. All cases of hypokalaemia will be reported as adverse events.

Study participants will receive non-pharmacological recommendations for stone prevention according to current nephrolithiasis guidelines at all study visits.^14 15^ Recommendations will include increased fluid intake with circadian drinking to ensure daily urinary volumes of at least 2–2.5 L, a balanced diet rich in vegetables and fibres with normal calcium content (1–1.2 g/day) but limited sodium chloride (4–6 g/day) and animal protein (0.8–1 g/kg/day) content.

### Criteria for withdrawal/discontinuation of participants and handling of dropouts

Criteria for treatment discontinuation or study discontinuation are listed in table 3.

**Table 3:** Criteria for early withdrawal/discontinuation of study participants

**Criteria for withdrawal/discontinuation**	
Study participants must be withdrawn from the study if the following occurs:	
1	At the participants’ own request
2	If, in the investigator’s opinion, continuation of the study would be harmful to the participant’s well-being
3	If the participant discontinues treatment before the assigned second treatment period is completed
4	Use of prohibited medication indicated during study (see table 2)
7	Severe hypokalaemia (plasma potassium <2.5 mmol/L)
8	Start of uric acid lowering therapy
9	Pregnancy

Participants lost to follow-up or withdrawn before two treatment periods are completed will be replaced by new participants to reach a final number of 99 patients completing the study. A study participant who discontinues study participation prematurely for any reason will be defined as a dropout if the participant has already been randomised. A study participant who terminates the study before randomisation will be regarded as a screening failure. Any samples and data collected until study withdrawal will remain coded for the analysis.

### Statistical analysis

#### Sample size calculation

The null hypothesis that there is no difference in change in urine relative supersaturation ratios with treatment of indapamide or chlorthalidone compared with hydrochlorothiazide will be tested against the alternative that indapamide or chlorthalidone is superior to hydrochlorothiazide in reducing at least one of the two urine supersaturations (relative supersaturation ratios of calcium oxalate and calcium phosphate).

The sample size calculation was based on the following assumptions:

- Allocation ratio: 1:1:1 (3-arm crossover design).
- Type I and II error rate: 0.1 (two-sided) and 0.2, respectively.
- Effect measure: Difference in means of urine relative supersaturation ratios of calcium oxalate and calcium phosphate.
- Target treatment effect: 20% reduction.
- Analysis approach: Repeated-measures model adjusted for baseline as implemented in the sampsi2 package in Stata.^50^
- Correction for dropouts: Patients lost or withdrawn will be accounted for by randomising additional patients.
- Correction for multiple testing: Due to the exploratory nature of the study, no adjustment for multiple testing is done.
- Expected mean values, SD and correlations (between baseline and follow-up) of relative supersaturation ratios calcium oxalate and calcium phosphate in the hydrochlorothiazide group were calculated from NOSTONE data,^31^ which had identical eligibility criteria compared with this trial:
  - Assumptions for relative supersaturation ratio of calcium oxalate: mean, 7.5; SD, 5.0; correlation, 0.4. The target effect results in a reduction of 1.5 in absolute terms.
  - Assumptions for relative supersaturation ratio of calcium phosphate: mean, 2.5; SD, 2.3; correlation, 0.5. The target effect results in a reduction of 0.5 in absolute terms.

The expected difference of 20% relative supersaturation ratio reduction with indapamide or chlorthalidone compared with hydrochlorothiazide was chosen because this would translate into a clinically meaningful reduction of recurrence risk. In a past dietary trial of 5 years duration, a 20% reduction of relative supersaturation ratio of calcium oxalate at 1 week compared with baseline was associated with a 16% reduction in recurrence (a composite of symptomatic or radiologic recurrence) during follow-up.^12^ Our (unpublished) post hoc analysis of NOSTONE revealed similar results: A 20% reduction of relative supersaturation ratio of calcium oxalate at 3 months compared with baseline was associated with a 15% reduction in recurrence (a composite of symptomatic or radiologic recurrence) during follow-up. Relative supersaturation ratios of calcium oxalate and calcium phosphate were also significantly associated with radiologic recurrence on CT scan in NOSTONE, the most sensitive recurrence parameter. A 20% lower baseline value of relative supersaturation ratios of calcium oxalate or calcium phosphate was associated with a 7.5% and 9% lower risk of radiologic recurrence during follow-up. Based on the methods and assumptions described above, the resulting total sample size for relative supersaturation ratios of calcium oxalate and calcium phosphate is 58 and 99 patients, respectively. To ensure sufficient power for both primary outcomes, the larger sample size is chosen for this trial.

#### Primary analysis

The primary analysis will compare indapamide and chlorthalidone to hydrochlorothiazide for the two primary outcomes, relative supersaturation ratios of calcium oxalate and calcium phosphate. Given the exploratory nature of the trial, no adjustment for multiple testing will be made.^51^

Linear mixed-effects models will be used to analyse the log-transformed outcomes. Fixed effects will include the log-transformed baseline value, treatment (categorical) and period (categorical), with a random intercept for each participant. Relative differences (ratios) between pairwise treatments will be calculated. The relative change from baseline to day 28 will be further estimated for each treatment. Patients who drop out before completing at least two treatment periods, with one being hydrochlorothiazide, will be censored at the last available visit and considered for the completed comparison. All others will not be considered in the primary analysis. No imputation will be performed for missing data.

#### Secondary analyses

Secondary continuous outcomes will be analysed using the same approach, with log transformation applied when necessary.

#### Exploratory analysis

Changes in urinary extracellular vesicle NaCl cotransporter abundance will be compared between treatments using ordinary linear regression models adjusted for treatment period. Correlation analyses between NaCl cotransporter changes in urinary extracellular vesicles and urinary calcium, citrate, relative supersaturation ratios of calcium oxalate and calcium phosphate and plasma potassium will be conducted using Pearson’s or Spearman’s correlation coefficients, depending on data distribution.

#### Safety analysis

Safety outcomes will be presented descriptively.

### Quality assurance and control

To ensure quality in study conduct and data collection, trained and qualified monitors will visit the study site. During these visits, all source documents and relevant data will be accessible for review, and monitors’ questions will be addressed on-site. Findings and observations will be documented in site visit reports and shared with the responsible stakeholders. In addition, accumulating data will be regularly monitored by central data monitors using manual and statistical methods. All monitoring activities will follow a predefined monitoring plan established before the study begins (first participant enrolment).

### Data management

This trial uses an electronic data capture system (secuTrial) for data collection, with electronic case report forms implemented digitally. The system will only be activated after successfully completing a formal testing procedure. All data entered into the electronic case report forms are stored on a Linux server within a dedicated Oracle database. Inselspital Bern is responsible for hosting both the electronic data capture system and the database. The server is housed in a secure, locked room with access restricted to system administrators. All data entered into the electronic case report form are encrypted and transferred to the database using Transport Layer Security. The sponsor will retain the Trial Master File, extracted data, metadata, and interim and final reports for a minimum of 20 years.

### Data availability statement

De-identified individual participant data generated during the study will be made available on reasonable request to qualified researchers, following publication of the primary results, and in accordance with institutional and ethical guidelines. Data will be shared after approval of a methodologically sound proposal and signing of a data access agreement.

### Protocol version

Version 4.0, 29November 2024 (see document ‘INDAPACHLOR Protocol V4’ under online supplemental material).

### Ethics and dissemination

The INDAPACHLOR trial will be carried out in accordance with the protocol and with the principles enunciated in the current version of the Declaration of Helsinki, the guidelines of Good Clinical Practice issued by the International Council for Harmonisation of Technical Requirements for Pharmaceuticals for Human Use, the Swiss Human Research Act and Swiss regulatory authority’s requirements. The responsible Ethics Commission and Swissmedic will receive safety and interim reports and will be informed about study stop/end in agreement with local requirements. The study was approved by the cantonal Ethics Commission Bern, Switzerland on 28 May 2024 (approval # 2024_00477). Approval by Swissmedic was obtained on 27 June 2024 (approval # 701824).

The INDAPACHLOR trial is registered at ClinicalTrials.gov (NCT06111885) and the Swiss National Clinical Trials Portal (SNCTP000006156). Patient recruitment started in December 2024, and at the time of submission, 28 participants have been recruited. The study will presumably end in June 2027, and first results are expected in the fourth quarter of 2027. No publications containing the results of this study have already been published or submitted to any journal.

## Supplementary material

10.1136/bmjopen-2025-101594 online supplemental file 1

10.1136/bmjopen-2025-101594 online supplemental file 2

10.1136/bmjopen-2025-101594 online supplemental file 3
