# Supplementary material for: Indapamide or chlorthalidone to reduce urine supersaturation for secondary prevention of kidney stones: protocol for a randomised, double-blind, cross-over trial (INDAPACHLOR)
Source: BMJ Open. 2025 Jun 16;15(6):e101594. doi: 10.1136/bmjopen-2025-101594 (PMC12314834; doi:10.1136/bmjopen-2025-101594)
Supplement: online supplemental file 1 [file bmjopen-15-6-s001.pdf]

Clinical Study Protocol

Indapamide or Chlorthalidone to Reduce Urine Supersaturation for Secondary Prevention of Kidney Stones: a Randomized, Double-blind, Crossover Trial

INDAPACHLOR Trial

Study Type & Categorisation according to the Swiss Clinical Trials Ordinance (ClinO):	Clinical trial with Investigational Medicinal Product (IMP),<br>Risk category B
Study Registration:	ClinicalTrials.gov, Swiss National Clinical Trials Portal (SNCTP)
Study Identifier:	INDAPACHLOR Trial
Sponsor:	Insel Gruppe AG<br>Freiburgstrasse 8<br>CH-3010 Bern, Switzerland<br><br>Responsible person:<br>Prof. Dr. med. Daniel Fuster<br>Leitender Arzt<br>Department of Nephrology and Hypertension<br>Inselspital, Bern University Hospital<br>Freiburgstrasse 11<br>CH-3010 Bern, Switzerland<br>Phone: + 41 (0) 31 632 31 44<br>Email: daniel.fuster@insel.ch
Investigational Medicinal Products:	Indapamide, Chlorthalidone (IMPs in testing)<br>Hydrochlorothiazide (comparator)
Protocol Version and Date:	Version 4.0, 29.11.2024

CONFIDENTIAL

The information contained in this document is confidential and the property of the sponsor. The information may not - in full or in part - be transmitted, reproduced, published, or disclosed to others than the applicable Competent Ethics Committee(s) and Regulatory Authority(ies) without prior written authorisation from the sponsor except to the extent necessary to obtain informed consent from those who will participate in the study.

Signature Page

Study Identifier:	INDAPACHLOR Trial
Study Title:	Indapamide or Chlorthalidone to Reduce Urine Supersaturation for Secondary Prevention of Kidney Stones: a Randomized, Double-blind, Crossover Trial

By signing below, the signees approve this protocol version and confirm hereby to conduct the study according to the protocol, the current version of the World Medical Association Declaration of Helsinki, the ICH-E6 (GCP) guidelines, and the applicable national legal and regulatory requirements.

Sponsor & Principal Investigator: Prof. Dr. med. Daniel Fuster

Daniel Fuster Digital unterschrieben von Daniel Fuster Datum: 2024.12.03 10:09:53 +01'00'

Place/Date Signature

Trial Statistician and Methodologist: Dr. Marie Roumet

Digitally signed by Roumet Marie
Date: 2024.12.03 09:26:54 +01'00'

Place/Date Signature

Table of Contents

STUDY SYNOPSIS **5**
ABBREVIATIONS **7**
REVISION HISTORY **8**
STUDY SCHEDULE **9**
1. STUDY ADMINISTRATIVE STRUCTURE **11**
1.1 Sponsor 11
1.2 Principal investigator 11
1.3 Statistician ("Biostatistician") 11
1.4 Laboratory 11
1.5 Monitoring institution 11
1.6 Independent data monitoring committee 11
1.7 Any other relevant committee, person, organisation, institution 11
2. ETHICAL AND REGULATORY ASPECTS **11**
2.1 Study registration 12
2.2 Categorisation of study 12
2.3 Competent ethics committee 12
2.4 Competent authorities 12
2.5 Ethical conduct of the study 12
2.6 Declaration of interest 13
2.7 Patient information and informed consent 13
2.8 Participant privacy and confidentiality 13
2.9 Early termination of the study 13
2.10 Protocol amendments 13
3. BACKGROUND AND RATIONALE **14**
3.1 Background and rationale 14
3.2 Investigational medicinal product (treatment) and indication 14
3.3 Preclinical evidence 15
3.4 Clinical evidence to date 15
3.5 Rationale for the dosage, route, regimen 15
3.6 Explanation for choice of comparator 16
3.7 Risks / benefits 16
3.8 Justification of choice of study population 17
4. STUDY OBJECTIVES **17**
4.1 Overall objective 17
4.2 Primary objective 17
4.3 Secondary objectives 17
4.4 Safety objectives 17
4.5 Exploratory objectives 17
5. STUDY OUTCOMES **17**
5.1 Primary outcome 17
5.2 Secondary outcomes 17
5.3 Other outcomes of interest 18
5.4 Safety outcomes 18

6. STUDY DESIGN ... 18
6.1 General study design and justification of design ... 18
6.2 Methods of minimising bias ... 19
6.3 Unblinding procedures (code break) ... 19
7. STUDY POPULATION ... 19
7.1 Eligibility criteria ... 19
7.2 Recruitment and screening ... 20
7.3 Assignment to study groups ... 20
7.4 Criteria for withdrawal / discontinuation of participants ... 20
8. STUDY INTERVENTION ... 21
8.1 Identity of investigational medicinal products ... 21
8.2 Administration of experimental and control interventions ... 21
8.3 Dose modifications ... 22
8.4 Compliance with study intervention ... 22
8.5 Data / sample collection and follow-up for withdrawn participants ... 22
8.6 Trial specific preventive measures ... 22
8.7 Concomitant interventions (treatments) ... 23
8.8 Study drug accountability ... 23
8.9 Return or destruction of study drug ... 23
9. STUDY ASSESSMENTS ... 24
9.1 Study flow chart(s) / table of study procedures and assessments ... 24
9.2 Assessments of outcomes ... 24
9.3 Procedures at each visit ... 25
10. SAFETY ... 28
10.1 Drug studies ... 28
11. STATISTICAL METHODS ... 30
11.1 Hypothesis ... 30
11.2 Determination of sample size ... 30
11.3 Statistical criteria of termination of trial ... 31
11.4 Planned analyses ... 31
11.5 Handling of missing data and drop-outs ... 32
12. QUALITY ASSURANCE AND CONTROL ... 32
12.1 Data handling and record keeping/archiving ... 32
12.2 Data management ... 33
12.3 Monitoring ... 33
12.4 Audits and inspections ... 34
12.5 Confidentiality, data protection ... 34
12.6 Storage of biological material and related health data ... 34
13. PUBLICATION AND DISSEMINATION POLICY ... 34
14. FUNDING AND SUPPORT ... 34
14.1 Funding ... 34
15. INSURANCE ... 34
16. REFERENCES ... 35
17. APPENDICES ... 38

STUDY SYNOPSIS

**Sponsor:**	Insel Gruppe AG<br>Responsible person: Prof. Dr. med. Daniel Fuster
**Study title:**	Indapamide or Chlorthalidone to Reduce Urine Supersaturation for Secondary Prevention of Kidney Stones: a Randomized, Double-blind, Crossover Trial
**Study ID:**	INDAPACHLOR Trial
**Protocol version and date:**	Version 4.0, 29.11.2024
**Trial registration:**	ClinicalTrials.gov, Swiss National Clinical Trials Portal (SNCTP)
**Study category and rationale**	Study category B according to ClinO Art. 19: Investigational Medicinal Products (IMP) that are not used within the same disease group as in the authorized indication. Indapamide and hydroychlorothiazide are authorised in Switzerland as single agent preparations. Chlorthalidone is only approved as a combination preparation. However, the study is not intended to extend the authorisation or launch a new chlorthalidone preparation.
**Clinical phase:**	Therapeutic, exploratory clinical study
**Background and rationale:**	Kidney stones are the most common condition affecting the kidney. Both prevalence and incidence are increasing rapidly, driven by global warming, urbanization, dietary habits and occupational changes. Kidney stones are highly recurrent, associated with increased mortality, significant morbidity and reduced quality of life, and result in enormous health care expenditures. Hence, effective preventive measures are an undisputed medical need. Thiazide and thiazide-like diuretics (“thiazides”) have been the cornerstone of pharmacologic recurrence prevention since >50 years. NOSTONE, the only state-of-the-art trial ever performed for pharmacologic recurrence prevention, recently revealed that the most widely prescribed and best studied thiazide, hydrochlorothiazide, is not effectively preventing kidney stone recurrence. If these results also apply to the two more potent and long-acting thiazide-like diuretics indapamide and chlorthalidone is currently unknown. No head-to-head comparison of different thiazides for prevention of kidney stone recurrence has ever been performed. Thus, the role of thiazides in the prevention of kidney stone recurrence remains unclear. This poses the urgent need for a clinical trial that addresses this critical knowledge gap.
**Objective(s):**	The primary objective is to determine if indapamide 2.5 mg daily or chlorthalidone 25 mg daily are superior to hydrochlorothiazide 50 mg daily in reducing urine Urine Supersaturations (RSRs) of Calcium Oxalate (CaOx) Calcium Phosphate (CaP) over a treatment period of 28 days as an indicator of the prophylactic potential.<br>Secondary objectives are to assess the impact of indapamide 2.5 mg daily, chlorthalidone 25 mg daily or hydrochlorothiazide 50 mg daily on supplementary urine and blood parameters including those to assess selected safety aspects.
**Outcome(s):**	The primary objective will be addressed by the following two outcomes:<br>– Change in RSR CaOx from baseline to day 28 of each treatment period.<br>– Change in RSR CaP from baseline to day 28 of each treatment period.<br>The secondary objectives will be addressed by assessing the change of blood and 24-hour urine parameters from baseline to day 28 days of each treatment period.
**Study design:**	Randomized, double-blind, crossover trial

**Inclusion / exclusion criteria:**	**Inclusion criteria**<br>– Written, informed consent.<br>– Age 18 years or older.<br>– Recurrent kidney stone disease (≥2 kidney stone episodes in the last 10 years prior to randomisation).<br>– Past kidney stone containing ≥50 % CaOx, CaP, or a mixture of both.<br>**Exclusion criteria**<br>– Patients with secondary causes of recurrent calcium kidney stones including severe eating disorders (anorexia or bulimia), chronic bowel disease, intestinal or bariatric surgery, sarcoidosis, primary hyperparathyroidism, chronic urinary tract infection.<br>– Patients with the following medications: Thiazide or loop diuretics, carbonic anhydrase inhibitors (including topiramate), xanthine oxidase inhibitors, alkali, active vitamin D (calcitriol or similar), calcium supplementation, bisphosphonates, denusomab, teriparatide, sodium-glucose co-transporter 2 (SGLT2) inhibitors, strong CYP3A4 inhibitors or inducers (may affect indapamide metabolism), lithium. (To be eligible for study participation, patients taking any of the above listed medications at screening must be willing to discontinue these medications at least 28 days before randomization.)<br>– Patients with chronic kidney disease (defined as CKD-EPI eGFR <30 mL/min).<br>– Patients with glomerulonephritis.<br>– Patients with the following biochemical imbalances: severe hypercalcemia (>2.8 mmol/L), therapy-resistant hypokalemia or conditions with increased potassium loss, severe hyponatremia (<130 mmol/L), symptomatic hyperuricemia.<br>– Patients with hepatic encephalopathy or severe liver insufficiency.<br>– Patients with severe cardiac insufficiency.<br>– Patient with a recent cerebrovascular event.<br>– Patients with a solid organ transplant.<br>– Pregnant and lactating women. (A urine pregnancy test must be performed for women of child-bearing potential, defined as women who are not surgically sterilized/hysterectomized, and/or who are postmenopausal for less than 12 months.)<br>– Previous (within 3 months prior to randomization) or concomitant participation in another interventional clinical trial.<br>– Previous participation in INDAPACHLOR.<br>– Inability to understand and follow the protocol.<br>– Allergy to any one of the study drugs.
**Measurements and procedures:**	Patients will be allocated to indapamide 2.5 mg once daily, chlorthalidone 25 mg once daily and hydrochlorothiazide 50 mg once daily in a random sequence. The three consecutive active treatment periods of 28 days each will be separated by wash-out periods of 28 to 56 days.<br>Variables analyzed for the primary outcome: RSR CaOx and RSR CaP at 28 days with indapamide or chlorthalidone compared to hydrochlorothiazide. RSRs will be calculated by the EQUIL2 program [1].<br>Variables analyzed for the secondary outcomes: 24-hour urine and blood parameters.<br>Variables analyzed for the safety outcomes: Vital signs, adverse events of special interest (see paragraph 9.2.4.2), Serious Adverse Events (SAEs).
**Study product / intervention:**	Oral chlorthalidone 25 mg once daily for 28 days and oral indapamide 2.5 mg daily for 28 days.
**Control intervention:**	Oral hydrochlorthiazide 50 mg for 28 days.

**Number of participants with rationale:**	124 individuals will be recruited in the study yielding 80 % power to detect a 20 % change in urinary RSR CaOx or CaP with chlorthalidone or with indapamide compared to hydrochlorothiazide, using a two-sided significance level of 0.1.
**Study duration (anticipated):**	06/2024-10/2028
**Study schedule (anticipated):**	Month/Year of First-Participant-In: 12/2024<br>Month/Year of Last-Participant-In: 11/2028<br>Month/Year of Last-Participant-Out: 04/2029
**Investigator(s):**	Principal investigator:<br>Prof. Dr. med. Daniel Fuster, Leitender Arzt<br>Department of Nephrology and Hypertension, Inselspital, Bern University Hospital<br>Freiburgstrasse 11, CH-3010 Bern, Switzerland<br>Email: daniel.fuster@insel.ch<br>Phone: +41 (0)31 632 31 44
**Study centre(s):**	Single-center: Inselspital, Bern University Hospital
**Statistical considerations:**	The primary analysis will include all randomized patients that completed at least two treatment periods. In a per-protocol analysis, only patients that completed all three periods and had no major protocol deviations will be considered. Due to the exploratory nature of the study, no adjustment for multiple testing will be done. False positive results will not alter practice. No interim analysis is foreseen.
**GCP statement:**	This study will be conducted in compliance with the protocol, the current version of the World Medical Association Declaration of Helsinki, ICH-E6 (GCP), and the applicable national legal and regulatory requirements.

ABBREVIATIONS

AE	Adverse Event
AESI	Adverse Events of Special Interest
BASEC	Business Administration System for Ethical Committees
CA	Competent Authority
CaOx	Calcium Oxalate
CaP	Calcium Phosphate
CEC	Competent Ethics Committee
ClinO	Clinical Trials Ordinance
CRF	Case Report Form (eCRF = electronic CRF)
CTCAE	Common Terminology Criteria for Adverse Events
DCR	Department of Clinical Research, University of Bern
EDC	Electronic Data Capture
EOS	End Of Study
EOT	End Of Treatment
GCP	Good Clinical Practice
HbA1c	Hemoglobin A1c
ICH	International Council for Harmonisation of Technical Requirements for Pharmaceuticals for Human Use
IMP	Investigational Medicinal Product
NCC	Sodium/Chloride Co-transporter

NIH	National Institute of Health
RCT	Randomized Controlled Trial
RSR	Relative Supersaturation Ratio
SmPC	Summary of Product Characteristics
SNCTP	Swiss National Clinical Trials Portal
SUSAR	Suspected Unexpected Serious Adverse Reaction
uEV	urinary Extracellular Vesicles

REVISION HISTORY

Version No. & Date	Chapter	Description of change	Reason for the change
1.0, 27.02.2024	N/A	N/A	N/A – first version (submitted to Ethics committee, not approved)
2.0, 17.04.2024	- Study Schedule<br>- Chap 2.3, 2.4<br>- Chap 8.5, 8.6.3<br>- Chap 9.3<br>- Chap 10.1.2<br>- Chap 11.2<br>- Chap 12.1.2	Adaptations following response from Ethics committee	Feedback received from Ethics committee
3.0, 12.07.2024	Main changes:<br>- Study Synopsis<br>- Chap 7.1<br>- Chap 8.6.2	Adaptations of exclusion criteria and prohibited medications based on the SmPC of the IMPs	Feedback received from Swissmedic (condition in “Verfügung” from Swissmedic from 27.06.2024)
	Diverse	Small changes to improve clarity; correction of discrepancies; adaptation of the anticipated study timelines in the synopsis	General revision
4.0, 29.11.2024	- Chap 2.3, 2.4, 2.5, 2.10<br>- Chap 10.1.2<br>- Chap 13	Adaptation to the revised Clinical Trials Ordinance (ClinO)	Revised ClinO that came into effect on 01.11.2024
	Diverse	Adaptation of the anticipated study timelines in the synopsis; standardisation of formatting; change of the responsible trial statistician	General revision

STUDY SCHEDULE

Study Period	Screening[1]		Treatment period 1			Treatment period 2			Treatment period 3			Safety follow-up
Visit	1 (🧍/☏)	2 (🧍)	3 (🧍) Baseline	4 (🧍)	5[5] (🧍)	6 (🧍)	7 (🧍)	8[5] (🧍)	9 (🧍)	10(🧍)	11 (🧍)	EOS (☏)
Visit time points	- 30 to - 15 days	-14 days to day 0	Day 1	Day +7	Day +28	Day +57	Day +64	Day +84	Day +113	Day +120	Day +140	30 days after EOT
Allowed visit window			0	+7 days	+ 7 days	0	+ 7 days	+ 7 days	0	+ 7 days	+ 7 days	± 7 days
Patient information & informed consent	x											
In-/exclusion criteria[11]	x	x	x									
Demographics	x											
Medical history	x											
Stone composition	x[2]											
Physical examination[9]	x		x		x	x		x	x		x	
Vital signs[3]	x		x		x	x		x	x		x	
Blood analysis[13]		x	x	x	x	x	x	x	x	x	x	
Urine analysis (24-hour collection)[4]		x	x		x	x		x	x		x	
Urine analysis (uEVs)[10]			x		x	x		x	x		x	
Blood/urine for biobank[6,12]			(x)		(x)	(x)		(x)	(x)		(x)	
Pregnancy test (urine)[7]			x			x			x			
Randomization			x									
AEs of special interest		x	x	x	x	x	x	x	x	x	x	x
SAEs and AEs related to SAEs		x	x	x	x	x	x	x	x	x	x	x
Concomitant medication	x	x	x		x	x		x	x		x	
Hand out of (new) IMP			x IMP 1			x IMP 2			x IMP 3			
Collection of used/unused IMP bottles					x			x			x	
Daily intake of IMP[8]				IMP 1			IMP 2			IMP 3		
Instruction on non-pharmacologic recommendations for stone prevention			x			x			x			

🚶, in-person; ✆, phone call; EOS, End Of Study; EOT, End Of Treatment

1 For participants in current follow-up for kidney stone disease, all assessments of visit 1 and visit 2 are performed at visit 3. In this case, patients will be informed by phone about the study and receive the written study information at least 14 days prior to visit 3. For participants with first time work-up for kidney stone disease (new referrals), screening assessments should be performed at visits 1 and 2, unless the distribution over 2 visits is impracticable for the patient, in which case the same procedure as for participants in current follow-up applies.
2 Information on the composition of all stones analyzed prior to randomization as available.
3 Heart rate, systolic and diastolic blood pressure at the right arm in sitting position after at least 5 minutes at rest.
4 The 24-hour urine collection during the screening period (visit 2) is part of the routine procedures (not study specific). 24-hour urine collections scheduled for visits 3, 5, 6, 8, 9, 11 will start 24 hours prior to the visits.
5 Visit is followed by a 28 day wash out. Wash out periods have to be at least 28 days but may be extended to maximally 56 days. If the wash out period is longer than 28 days, all subsequent study visits will be adapted accordingly.
6 Biobank samples will only be collected in patients that provided the specific consent for this.
7 For women of child-bearing potential (defined as women who are not surgically sterilized/hysterectomized, and/or who are postmenopausal for less than 12 months), a pregnancy test (from urine) will be performed at the begin of each active treatment period before the start of the IMP. Women of child-bearing potential must use an effective contraceptive during treatment with IMP (see paragraph 8.6.3).
8 Daily intake of IMP starts the day of the visit, after blood draw and collection of second morning fasting urine and continues until the day of the next visit. If the patient is taking a co-medication listed in the exclusion criteria, a washout period of 28 days has to be done prior to IMP start.
9 General physical examination including body weight and height (height will only be measured once at screening or baseline).
10 Second morning urine collections will be made after at least 6 hours fasting. Fasting second morning urine collection will be made after the end of the 24-hour urine collection.
11 At visit 1, pre-assessment of eligibility based on data available per routine, at visits 2 and 3, full assessment of eligibility.
12 Blood collection for the biobank will be made after at least 6 hours fasting. Urine collection for the biobank will be taken from the 24-hour urine collection.
13 Blood collections at visit 2, 3, 5, 6, 8, 9 and 11 will be made after at least 6 hours fasting. No fasting is required for blood collections at visits 4, 7, and 10 (plasma potassium only).

1. STUDY ADMINISTRATIVE STRUCTURE

1.1 Sponsor

Insel Gruppe AG
Freiburgstrasse 8
CH-8010 Bern, Switzerland

Responsible person:
Prof. Dr. med. Daniel Fuster, Leitender Arzt
Department of Nephrology and Hypertension, Inselspital, Bern University Hospital
Freiburgstrasse 11, CH-3010 Bern, Switzerland
Email: daniel.fuster@insel.ch
Phone: +41 (0)31 632 31 44

1.2 Principal investigator

Same as sponsor responsible person (see paragraph 1.1)

1.3 Statistician ("Biostatistician")

Dr. Marie Roumet
Department of Clinical Research (DCR), University of Bern
Mittelstrasse 43, CH-3012 Bern, Switzerland
Email: marie.roumet@unibe.ch
Phone: +41 (0)31 684 56 74

1.4 Laboratory

ZLM (Zentrum für Labormedizin)
Inselspital, Bern University Hospital
CH-3010 Bern, Switzerland
Phone: +41 (0)31 632 22 01

1.5 Monitoring institution

Department of Clinical Research (DCR), University of Bern
Mittelstrasse 43, CH-3012 Bern, Switzerland
Phone: +41 (0)31 684 33 72

1.6 Independent data monitoring committee

Not applicable.

1.7 Any other relevant committee, person, organisation, institution

Medical expert: Same as sponsor responsible person (see paragraph 1.1)

2. ETHICAL AND REGULATORY ASPECTS

Before the study will be conducted, the protocol, the proposed participant information and consent form, as well as other study-specific documents shall be submitted to a properly constituted Competent Ethics

Committee (CEC) and Competent Authority (CA, Swissmedic) in agreement with local legal requirements, for formal approval. Any amendment to the protocol must as well be approved (if legally required) by these institutions.

The decision of the CEC and CA concerning the conduct of the study will be made in writing to the sponsor before commencement of this study. The study can only begin once approval from all required authorities has been received. Any additional requirements imposed by the authorities shall be implemented.

2.1 Study registration

Upon approval, the study will be registered in the Clinical Trials Registry Platform of the National Institute of Health (NIH) – ClinicalTrials.gov. In addition, the trial will be registered in the Swiss National Clinical Trials Portal (SNCTP).

2.2 Categorisation of study

Risk category B according to ClinO Art. 19: Clinical trial with Investigational Medicinal Products (IMP) that are not used within the same disease group as in the approved indication. Indapamide and hydroychlorothiazide are authorised in Switzerland as single agent preparations. Chlorthalidone is only approved as a combination preparation. However, the study is not intended to extend the authorisation or launch a new chlorthalidone preparation.

2.3 Competent ethics committee

The sponsor ensures that approval for the clinical trial from the CEC is sought before the start of the trial through the Business Administration System for Ethics Committees (BASEC) portal (https://submissions.swissethics.ch/en/).

No changes will be made to the protocol without prior consultation of the sponsor and, in case of significant amendments according to ClinO Art. 29, CEC approval. Exempt from this requirement are measures that have to be taken immediately in order to protect the study participants. Any safety or protective measures that have to be taken during the conduct of the trial due to unanticipated risks to humans will be reported to the CEC within 7 days. Amendments will be reported according to paragraph 2.10, and intermediary reports (annual safety reports) will be forwarded to the CEC yearly.

The investigator reports the premature termination, interruption, or resumption of the study, including the reasons thereof, to the CEC within 15 days. An interruption lasting more than two years is considered a premature termination. The investigator reports the first visit of the first participant and the end of the study (defined as the last visit of the last participant) to the CEC within 30 days. The final study report will be submitted within one year after the completion of the study or premature study termination.

2.4 Competent authorities

The sponsor will obtain approval from the CA (Swissmedic) before the start of the trial.

No changes will be made to the protocol or in research activity without prior sponsor approval, and, in case of significant changes according to ClinO Art. 34, CA approval. Exempt from this requirement are measures that have to be taken immediately in order to protect the study participants. Any safety or protective measures that have to be taken during the conduct of the study due to unanticipated risks to humans will be reported to the CA within 7 days. Amendments will be reported according to paragraph 2.10, and intermediary reports (annual safety reports) will be forwarded to the CA yearly.

The investigator reports the premature termination, interruption, or resumption of the study, including reasons thereof, to the CA within 15 days. An interruption lasting more than two years is considered a premature termination. The investigator reports the first visit of the first participant and the end of the study to the CA within 30 days. The final study report will be submitted within one year after the completion of the study or premature study termination.

2.5 Ethical conduct of the study

The study will be carried out in accordance with the protocol and with the principles enunciated in the current version of the Declaration of Helsinki, the guidelines of Good Clinical Practice (GCP) issued by the International Council for Harmonisation of Technical Requirements for Pharmaceuticals for Human Use (ICH), the Swiss Law, and Swiss regulatory authority's requirements. The CEC and CA will receive annually safety and interim reports and will be informed about study stop/end in agreement with local

requirements. The CEC will also be informed annually about the general progress of the clinical trial.

2.6 Declaration of interest

The study will be supported by a research grant of the Medical Faculty of the University of Bern and the Inselspital, Bern University Hospital.

2.7 Patient information and informed consent

Candidates may be informed of the study by phone or in person. The investigator will explain the following to each potential participant:

- the nature of the study, its purpose, the procedures involved, the expected duration, the potential risks and benefits, and any discomfort it may entail
- that participation in the study is voluntary and that they may withdraw from the study at any time
- that withdrawal of consent will only terminate study related treatment and investigations, but not affect their subsequent medical assistance and treatment
- that the participant's medical records may be examined by authorised individuals other than their treating physician

In case potential participants show interest in the study, they will receive a participant information sheet and a consent form describing the study and providing sufficient information and time (at least 1 week) for them to make an informed decision about whether to participate in the study. The participant information sheet and consent form may either be handed out to the candidate at the occasion of a (regular) visit at the site or it may be sent by postal or electronic mail prior to the potential screening visit. The candidate should read and consider the statement and have the possibility for a discussion with the investigator in which all open questions are resolved before signing and dating the informed consent form. The consent form must also be signed and dated by the investigator (or his designee) who has discussed the study with the participant, and it will be retained as part of the study records. A copy of the signed document will be provided to each participant.

The formal consent of a participant, using the approved consent form, must be obtained before the participant is submitted to any study procedure. Patients are asked to sign an additional informed consent form to allow collection of additional samples for storage in the INDAPACHLOR biobank and potential use in future research projects.

2.8 Participant privacy and confidentiality

The investigator affirms and upholds the principle of the participant's right to privacy and that they comply with applicable privacy laws. Especially, anonymity of the participants shall be guaranteed when presenting the data at scientific meetings or publishing them in scientific journals. Individual subject medical information obtained as a result of this study is considered confidential and disclosure to third parties is prohibited. Subject confidentiality will be further ensured by utilising subject identification code numbers to correspond to treatment data in the computer files. For study-related monitoring, audits, CEC review, and CA inspections, the investigator will provide direct access to source data and source documents, including parts of the medical records relevant to the study (e.g. medical history) to authorised representatives of the sponsor, the CA, or CEC.

2.9 Early termination of the study

The sponsor may terminate the study prematurely according to certain circumstances, for example:

- ethical concerns,
- insufficient participant recruitment,
- when the safety of the participants is doubtful or at risk, respectively,
- alterations in accepted clinical practice that make the continuation of a clinical trial unwise,
- early evidence of benefit or harm of the experimental intervention.

Premature study end or interruption of the study will be reported to the CEC and CA according to paragraphs 2.3 and 2.4.

2.10 Protocol amendments

Significant amendments are only implemented after approval of the CEC and CA respectively. Under

emergency circumstances, deviations from the protocol to protect the rights, safety, and well-being of human subjects may proceed without prior approval of the sponsor and the CEC/CA. Such deviations must be documented and reported to the sponsor within 24 hours and to the CEC/CA within 7 days. All non-significant amendments are communicated to the CA as soon as possible if applicable and once a year to the CEC with the safety report / general study progress report of the trial.

3. BACKGROUND AND RATIONALE

3.1 Background and rationale

Nephrolithiasis is by far the most common kidney disease, affecting up to 20 % of men and 10 % of women worldwide [2]. Both prevalence and incidence of kidney stones increased in recent decades, irrespective of age, sex, and ethnicity [3, 4]. Kidney stones are highly recurrent, extremely painful and cause enormous cost, excess morbidity and reduced quality of life [5-7]. Thus, prevention of kidney stone recurrence is of critical importance [8-10].

Most kidney stones are composed of Calcium Oxalate (CaOx), Calcium Phosphate (CaP), or a mixture of both. High urine calcium (hypercalciuria) is the most frequent metabolic abnormality among patients with kidney stones [11]. Yet, urine calcium is not a dichotomous risk factor for kidney stone formation. An increased risk for kidney stone formation was observed when urine calcium excretion exceeded 5 mmol (=200 mg)/24 hours, which is below the current definition of “hypercalciuria” (>7.5 mmol (=300 mg)/24 hours in men and >6.25 mmol (=250 mg)/24 hours in women) [12].

Supersaturation, the presence of a salt in solution at a concentration exceeding its own solubility, is the driving force for crystallization and therefore kidney stone formation. At a supersaturation <1, crystals dissolve, at a supersaturation >1, crystals form [13]. Relevant urine supersaturations for the formation of calcium-containing kidney stones are CaOx and brushite, a form of CaP [14]. Urine calcium drives supersaturation for both, CaOx and CaP, and thus increases the risk for the formation of CaOx and CaP stones [13]. Urine supersaturations are calculated using a set of 14 biochemical parameters measured in 24-hour urines by a dedicated software, EQUIL2 (usually referred to as Relative Supersaturation Ratio – RSR) [1]. Urine supersaturations are highly correlated with kidney stone composition and well established proxies for the risk of recurrent stone formation [14-18]. Interventions that reduced stone events in Randomized Controlled Trials (RCTs) and prospective studies closely correlated with reductions in urine RSRs calculated with EQUIL2 [15, 16, 19, 20].

Thiazide and thiazide-like diuretics (“thiazides”) have been the cornerstone of pharmacologic recurrence prevention since >50 years. NOSTONE, the only state-of-the-art trial ever performed for pharmacologic recurrence prevention, recently revealed that the most widely prescribed and best studied thiazide, hydrochlorothiazide, is not effectively preventing kidney stone recurrence [21]. If these results also apply to the two more potent and long-acting thiazide-like diuretics indapamide and chlorthalidone is currently unknown. No head-to-head comparison of different thiazides for kidney stone recurrence prevention or for the established biochemical proxies of recurrence risk, urine RSR CaOx and CaP, has ever been performed. Therefore, a definitive statement on the effectiveness of thiazides in general for kidney stone recurrence prevention is currently not possible. This critical knowledge gap creates the need for a randomized trial that directly compares the efficacy of different thiazides for the prevention of kidney stone recurrence.

3.2 Investigational medicinal product (treatment) and indication

Thiazide diuretics reduce urine calcium excretion (and hence RSR CaOx and CaP), and their purported efficiency in recurrence prevention has been mainly attributed to this unique property. Reduction of urine citrate is a well-known side effect of thiazides, that counteracts reductions in RSR CaOx and CaP induced by thiazide-induced reduction of urine calcium. The mechanism is thought to be thiazide-induced hypokalemia that results in intracellular acidosis, stimulating avid citrate reabsorption in the proximal tubule.

The lack of efficacy of hydrochlorothiazide in NOSTONE, recent concerns about skin cancer associated with long-term use of hydrochlorothiazide [22-24], coupled with the absence of effective and well-tolerated alternatives for recurrence prevention, will inevitably lead to a prescription shift to the two thiazide-like diuretics indapamide and chlorthalidone, which are more potent and have a significantly longer half-life compared to hydrochlorothiazide.

We conducted a systematic literature search on the impact of indapamide or chlorthalidone on urine

calcium, urine citrate, and urine RSRs CaOx and CaP (Table 1). Indapamide at daily doses of 1.5 – 2.5 mg resulted in 20 – 56 % reductions of urine calcium compared to baseline without a clear dose-response effect. For chlorthalidone at daily doses of 25 – 100 mg, reductions of urine calcium of 22 – 56 % compared to baseline have been reported without a clear dose-response effect. These values exceed urine calcium reductions observed with hydrochlorothiazide in NOSTONE (9 - 17 % compared to baseline, 15-16 % compared to placebo) and past hydrochlorothiazide trials (20 - 40 %). With respect to urine citrate, no change up to a reduction of 32 % compared to baseline has been reported with indapamide without a clear dose-response effect. With 25 mg chlorthalidone daily, a reduction of urine citrate of 19 % compared to baseline has been reported in a single study. In patients receiving high doses of hydrochlorothiazide in NOSTONE (25 mg or 50 mg once daily), urine citrate tended to be lower compared to baseline and to patients receiving placebo, but differences were not significant. We found no data on the effects of chlorthalidone on RSRs in humans. For indapamide, a daily dose of 2.5 mg induced a 54 % reduction in RSR CaOx and a 22 % reduction in RSR CaP compared to baseline. In patients receiving hydrochlorothiazide in NOSTONE, there were no significant reductions in RSR CaOx and RSR CaP compared to baseline and patients receiving placebo.

Thus, indapamide and chlorthalidone seem to be more effective in reducing urine calcium than hydrochlorothiazide. However, due to the large variability of urine citrate data reported, the paucity of supersaturation data and the lack of direct comparisons, it remains unclear if indapamide and chlorthalidone are superior to hydrochlorothiazide in reducing RSR CaOx and CaP.

Table 1. *Impact of Indapamide and Chlorthalidone on urine composition.*

Variable	Dosage (mg/day)	Comparison	Exposure	Mean absolute change	Mean percent change	Author, Year
**Indapamide**						
RSR CaOx	2.5 mg	Baseline	36 months (m)	−4.08	−54 %	Borghi, 1993[25]
RSR CaP	2.5 mg	Baseline	36 m	−0.78	−61 %	Borghi, 1993[25]
Urine calcium (mmol/24h)	1.5 mg	Baseline	6 to 18 m	−4.90 to −4.55	−53 to −50 %	Alonso, 2012[26]
	2.5 mg	Baseline	3 m	−2.69	−44 %	Martins, 1996[27]
	2.5 mg	Baseline	6 to 36 m	−4.64 to −3.74	−48 to −39 %	Borghi, 1993[25]
	2.5 mg	Baseline & placebo	7 days	−2.70 to −1.80	−44 to −35 %	Borghi, 1988[28]
	2.5 mg	Baseline	3 m	−6.86 to −6.46	−56 to −52 %	Lemieux, 1986[29]
Urine citrate (mmol/24h)	1.5 mg	Baseline	6 to 18 m	−1.31 to −0.81	−32 to −20 %	Alonso, 2012[26]
	2.5 mg	Baseline	6 to 36 m	−0.10 to +0.04	−3.9 to +1.5 %	Borghi, 1993[25]
	2.5 mg	Baseline	3 m	−0.01	−1%	Martins, 1996[27]
**Chlorthalidone**						
RSR CaOx	Not available	Not available	Not available	Not available	Not available	Not available
RSR CaP	Not available	Not available	Not available	Not available	Not available	Not available
Urine calcium (mmol/24h)	25 mg	Baseline	6 m	−4.62	−42 %	Wolfgram, 2013[30]
	50-100 mg	Baseline	3 m	−3.99 to −1.95	−56 to −29 %	Coe, 1988[31]
	25-50 mg	Baseline	36 m	−1.87 to −1.65	−28 to −22 %	Ettinger, 1988[32]
	25-50 mg	Baseline	34 m	Not available	−36 %	Lockefeer, 1977[33]
Urine citrate (mmol/24h)	25 mg	Baseline	6 months	−0.92	−19 %	Wolfgram, 2013[30]

3.3 Preclinical evidence

There is no animal model, which recapitulates the pathophysiology of nephrolithiasis in humans. Hence, there is no supportive preclinical evidence for this trial.

3.4 Clinical evidence to date

Hydrochlorothiazide, indapamide, and chlorthalidone are all guideline recommended thiazides for kidney stone recurrence prevention and hence widely used for this indication [34-36]. The current state of evidence with respect to the individual efficacy of these three thiazides in preventing kidney stone recurrence is unclear and reviewed in paragraphs 3.1 and 3.2.

3.5 Rationale for the dosage, route, regimen

The three active treatments are indapamide 2.5 mg, chlorthalidone 25 mg, or hydrochlorothiazide 50

mg once daily per os in the morning with no dose modifications possible. Doses of indapamide and chlorthalidone we plan to use are supported by one trial each for recurrence prevention and are doses typically used in clinical routine for recurrence prevention [25, 32]. While indapamide doses exceeding 2.5 mg daily are not used clinically, chlorthalidone doses exceeding 25 mg daily are occasionally employed. In the RCT performed by Ettinger et al. [32], chlorthalidone 50 mg daily seemed to be equally effective in preventing recurrence as 25 mg daily, but was associated with a higher rate of adverse events. As comparator, we will use the highest dose of hydrochlorothiazide used in NOSTONE, 50 mg daily, which is considered to be equal to chlorthalidone 25 mg or indapamide 2.5 mg daily for blood pressure reduction and induction of biochemical changes in the blood that are hallmarks of thiazide action (increase of uric acid, decrease of potassium) [37-39].

To hold promise for kidney stone recurrence prevention, indapamide or chlorthalidone once daily need to display superior efficacy compared to hydrochlorothiazide once daily in reducing urine RSRs. Effects of hydrochlorothiazide 50 mg once daily as compared to placebo for kidney stone prevention are known as part of the NOSTONE results [21]. We will analyze relative changes between different thiazide treatments and from baseline, hence an additional placebo treatment period is not required in this trial. Duration of active treatment and wash out periods (28 days each) was chosen to ensure stable steady-state plasma concentrations for all three thiazides, of which chlorthalidone has the longest half-life, up to 60 hours with long term administration. For determination of active treatment and wash out duration, we additionally considered the fact that the pharmacodynamic response of thiazides is significantly longer than predicted by half-life [40].

3.6 Explanation for choice of comparator

Explanation for choice of the comparator hydrochlorothiazide has been reviewed in detail in paragraphs 3.4. and 3.5.

Furthermore, dietary and lifestyle interventions are highly effective in preventing recurrence in calcareous nephrolithiasis and are thus always the basis of stone prevention. As such, any trial assessing pharmacologic interventions for the prevention of stone recurrence must do so in the setting of state-of-the-art non-pharmacologic interventions. On the basis of RCT evidence, current guidelines [34-36] recommend the following non-pharmacologic measures in adult patients with recurrent calcareous nephrolithiasis: increased fluid intake with circadian drinking to ensure daily urinary volumes of at least 2 – 2.5 L, a balanced diet rich in vegetables and fibers with normal calcium content (1-1.2 g/day) but limited NaCl (4-5 g/day) and animal protein (0.8-1 g/kg body weight/day) content. Furthermore, patients must be advised to retain a normal Body Mass Index, have adequate physical activity and balance excessive fluid loss. All patients in this study will be instructed about these current non-pharmacologic recommendations for stone prevention.

3.7 Risks / benefits

Kidney stones are the most common condition affecting the kidney. Both prevalence and incidence are increasing rapidly, driven by global warming, urbanization, dietary habits, and occupational changes. Kidney stones are highly recurrent, associated with increased mortality, significant morbidity, and reduced quality of life, and result in enormous health care expenditures. Hence, effective preventive measures are an undisputed medical need. Yet, there are very limited treatment options available at the moment, and the efficacy of thiazides, the current standard medical treatment, remains unclear.

Indapamide, chlorthalidone, and hydrochlorothiazide are all approved in Switzerland for the treatment of arterial hypertension. Furthermore, all three thiazides are guideline-recommended for the indication explored in this study: kidney stone recurrence prevention [34-36]. Indapamide, chlorthalidone, and hydrochlorothiazide are in use worldwide since many decades, treatment risks are well characterized and manageable. Main adverse events observed include arterial hypotension, hypokalemia, gout, and new onset diabetes mellitus. Patient information and monitoring will be implemented to mitigate potential risks in the study population. Possible Adverse Events (AEs) should be considered in light of potential benefits including reduced risk of recurrence. However, since active treatment phases will only be 28 days each, benefits and risks of patients participating in the trial will likely both be minimal.

The results of the study will yield important clinical information for the care of patients with recurrent kidney stones, which is one of the most frequent human diseases (~10 % of worldwide population affected) and may lay the basis for a future RCT outcome trial. No competing trials are known.

3.8 Justification of choice of study population

Eligibility criteria were chosen to select for patients suffering from recurrent idiopathic calcium-containing kidney stones that would be treated with a thiazide for recurrence prevention based on current guidelines. All patients will undergo treatment with indapamide, chlorthalidone, and hydrochlorothiazide in a random sequence without stratification. No vulnerable individuals will be included in the study.

4. STUDY OBJECTIVES

4.1 Overall objective

The purpose of this study is to assess if indapamide or chlorthalidone are promising candidate compounds for reducing the risk of kidney stone recurrence to decide whether one (or both) is worthwhile to be evaluated in a large randomized-controlled trial with long-term follow-up and clinically relevant endpoint.

4.2 Primary objective

The primary objective is to determine if indapamide or chlorthalidone are superior to hydrochlorothiazide in reducing urine RSR CaOx and RSR CaP, the two best validated biochemical indicators of kidney stone recurrence, over a treatment period of 28 days.

4.3 Secondary objectives

Assessment of the impact of indapamide 2.5 mg daily, chlorthalidone 25 mg daily, or hydrochlorothiazide 50 mg daily on supplementary urine and blood parameters including those to assess selected safety aspects.

4.4 Safety objectives

Description of the safety of the interventions in the context of this study (taking into account that sample size and study duration do not allow for a conclusive safety profiling). To this end vital signs, adverse events of special interest, and serious adverse events will be collected, analyzed, and reported descriptively.

4.5 Exploratory objectives

To obtain a mechanistic understanding of potential effects observed with the three thiazides by analyzing the abundance of the Sodium/Chloride Co-transporter (NCC) in urinary extracellular vesicles (uEVs) at day 28 of treatment with either one of the three thiazides.

5. STUDY OUTCOMES

5.1 Primary outcome

The primary objective will be addressed by evaluating two primary outcomes. Both outcomes will be assessed separately as they reflect different mechanisms and are both of relevance to assess the prophylactic potential:

- Change in RSR CaOx from baseline to day 28 of each treatment period.
- Change in RSR CaP from baseline to day 28 of each treatment period.

RSRs will be calculated by the EQUIL2 program upon measurement of the relevant parameters in 24-hour urine [1].

5.2 Secondary outcomes

Assessment of the following variables relevant to the secondary outcomes:

Blood parameters: Sodium, potassium, chloride, calcium, magnesium, phosphate, creatinine, urea, uric acid, venous blood gas, PTH, 25-OH Vitamin D, 1,25-OH Vitamin D, glucose, hemoglobin A1c (HbA1c): Change from baseline to day 28 of each treatment period.

24-hour urine parameters: Sodium, potassium, chloride, calcium, magnesium, phosphate, creatinine,

urea, uric acid, oxalate, citrate, sulfate, ammonium, bicarbonate, pCO2, pH, volume: Change from baseline to day 28 of each treatment period.

5.3 Other outcomes of interest

Exploratory outcomes: Abundance of total and phosphorylated NCC in uEVs: Change from baseline to day 28 of each treatment period.

INDAPACHLOR Biobank: During the scheduled follow-up visits, we will collect plasma, serum, urine, and DNA samples of patients. Samples will be stored in the INDAPACHLOR Biobank at the Bern University Hospital Switzerland. Patients must sign a separate patient informed consent form for the INDAPACHLOR Biobank. The rules and procedures of the INDAPACHLOR Biobank are described in a specific booklet (Reglement der Biobank für die INDAPACHLOR Studie).

5.4 Safety outcomes

Safety of the intervention in the context of the study will be collected, analysed, and reported descriptively based on vital signs, adverse events of special interest (clinical ones and selected parameters from above), and serious adverse events.

6. STUDY DESIGN

6.1 General study design and justification of design

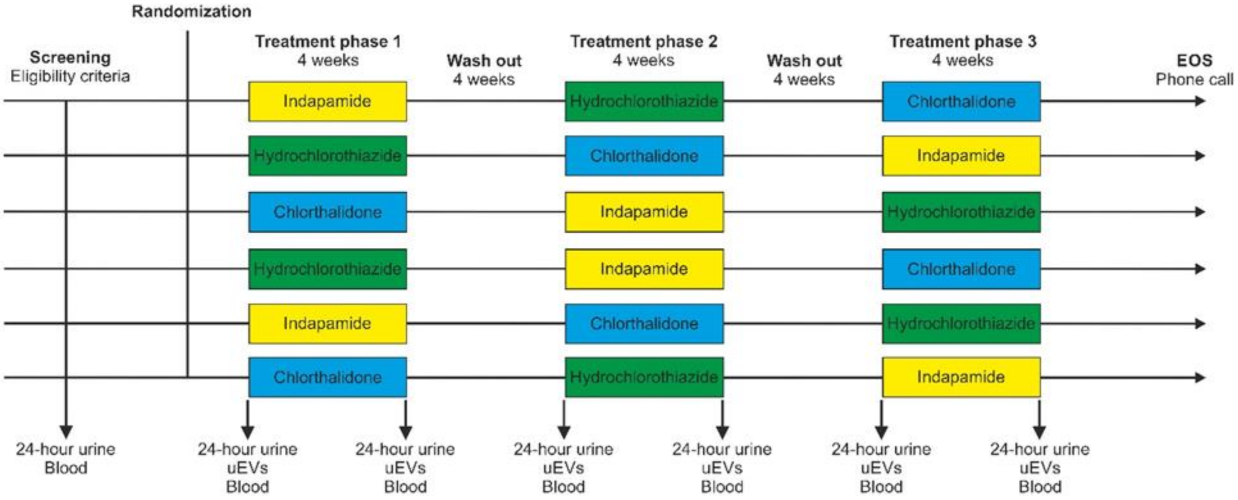

Figure 1. *uEVs: urinary extracellular vesicles. EOS: End Of Study*.

INDAPACHLOR is designed as a single-center, prospective, randomized, double-blind, crossover trial with three consecutive active treatment periods of 4 weeks each, separated by wash-out periods of 4 weeks after each active treatment period (Figure 1). We chose a uniform and balanced cross-over design with three treatments, three periods, and six sequences to avoid period, sequence, and first-order carry-over effects.

The study will enroll adult individuals with recurrent calcium-containing kidney stones with a history of at least 2 kidney stone events in the 10 years prior to randomization, defined as either spontaneous stone passage or urological removal of a stone. Eligible individuals will be randomized in equal proportions to one of six treatment sequences (Figure 1). If at the moment of informed consent signature, a patient is taking one or more of the concomitant medications listed as exclusion criterion in paragraph 7.1, these need to be stopped and a 28 days wash out period is required prior to randomization. During screening (as part of clinical routine), and during the study at the beginning of each active treatment phase (before starting intake of the IMP), and at the end of each active treatment phase, a 24-hour urine, a fasting morning blood draw and a second morning fasting urine sample (for uEVs) will be obtained. Active treatments phases will last 28 days and are separated by wash-out phases of 28 days.

6.2 Methods of minimising bias

6.2.1 Randomisation

All patients will undergo treatment with indapamide, chlorthalidone, and hydrochlorothiazide in a random sequence without stratification. Allocation will be concealed using sequentially coded drug bottles. Preparation and handling of the drug bottles will be done at a facility otherwise not involved in the trial (Apotheke Dr. Hysek AG, Bözingenstrasse 162, CH-2506 Biel, Switzerland). Randomization lists will be generated and stored at DCR with no access for persons directly involved in the trial. An independent data manager at DCR will communicate directly with Apotheke Dr. Hysek AG, which prepares the drug bottles.

6.2.2 Blinding procedures

Hydrochlorothiazide, indapamide, and chlorthalidone will be provided as identically looking capsules. Besides the consecutive number, bottles with capsules will look identical. All trial personnel but the data manager at DCR preparing the randomization list and the responsible person at Apotheke Dr. Hysek AG preparing the drug bottles will be blinded to the assigned treatment. Therefore, all trial personnel that is involved in recruitment and care of patients, trial assessment, monitoring, and analyses will be blinded to the assigned trial arm. Blinding will remain in place until the statistician codes the primary analysis of the primary and secondary outcomes and produces a dummy report of the primary analysis using a randomly generated group variable. The true group variable becomes open after the completion of the dummy report and gives place to the final report of all the analyses as well as the quality control by the independent statistician.

6.2.3 Other methods of minimising bias

No other methods of minimising bias used.

6.3 Unblinding procedures (code break)

Hydrochlorothiazide, indapamide, and chlorthalidone are approved drugs and have been in use for decades. Thus, their safety profiles are well known. Unblinding will only be allowed in situations where knowledge of the allocation is needed for the care of a patient. Because serious adverse reactions will be treated independently from any knowledge of treatment assignment, we do not expect any emergency unblinding. Note that a break of the randomization code per se is not a reason to stop study treatment or to withdraw the participant from the study.

Envelops with the treatment allocation will be prepared by Apotheke Dr. Hysek AG based on the randomization list generated by DCR personnel not otherwise involved in the study and stored at Apotheke Dr. Hysek AG (see paragraph 6.2.2). Unblinding will be conducted by phone through the principal investigator contacting the personnel on duty at Apotheke Dr. Hysek AG.

7. STUDY POPULATION

7.1 Eligibility criteria

Patients fulfilling all of the following <u>inclusion criteria</u> are eligible for the study:

- Written, informed consent.
- Age 18 years or older.
- Recurrent kidney stone disease (≥2 kidney stone episodes in the last 10 years prior to randomisation).
- Past kidney stone containing ≥50 % CaOx, CaP, or a mixture of both.

The presence of any one of the following <u>exclusion criteria</u> will lead to exclusion:

- Patients with secondary causes of recurrent calcium kidney stones including severe eating disorders (anorexia or bulimia), chronic bowel disease, intestinal or bariatric surgery, sarcoidosis, primary hyperparathyroidism, chronic urinary tract infection.
- Patients with the following medications: Thiazide or loop diuretics, carbonic anhydrase inhibitors (including topiramate), xanthine oxidase inhibitors, alkali, active vitamin D (calcitriol or similar), calcium supplementation, bisphosphonates, denusomab, teriparatide, sodium-glucose co-transporter 2 (SGLT2) inhibitors, strong CYP3A4 inhibitors or inducers (may affect

indapamide metabolism), lithium. (To be eligible for study participation, patients taking any of the above listed medications at screening must be willing to discontinue these medications at least 28 days before randomization.)

- Patients with chronic kidney disease (defined as CKD-EPI eGFR <30 mL/min).
- Patients with glomerulonephritis.
- Patients with the following biochemical imbalances: severe hypercalcemia (>2.8 mmol/L), therapy-resistant hypokalemia or conditions with increased potassium loss, severe hyponatremia (<130 mmol/L), symptomatic hyperuricemia.
- Patients with hepatic encephalopathy or severe liver insufficiency.
- Patients with severe cardiac insufficiency.
- Patient with a recent cerebrovascular event.
- Patients with a solid organ transplant.
- Pregnant and lactating women. (A urine pregnancy test must be performed for women of child-bearing potential, defined as women who are not surgically sterilized/hysterectomized, and/or who are postmenopausal for less than 12 months.)
- Previous (within 3 months prior to randomization) or concomitant participation in another interventional clinical trial.
- Previous participation in the INDAPACHLOR Trial.
- Inability to understand and follow the protocol.
- Allergy to any one of the study drugs.

7.2 Recruitment and screening

Eligibility criteria were chosen to select for patients suffering from recurrent idiopathic calcium-containing kidney stones that would be treated with a thiazide for recurrence prevention based on current guidelines.

Two different groups of individuals will be screened and recruited in the study by local investigators:

1) individuals newly referred for metabolic work-up for recurrent stone disease to study sites and
2) individuals who already underwent work-up for recurrent stone disease and are currently undergoing regular follow-up for recurrent stone disease at the study site.

After approval by the CEC and CA, the study will be announced to the internal Nephrology and Urology staff of Inselspital Bern. We will also inform external Nephrologists, Urologists, Internists, and Family practitioners by email upon approval by the CEC and CA. If available medical history indicates that an individual may be eligible for study participation, the individual will be informed in detail about the study by a study investigator. Inclusion in the study will take place only upon receipt of written informed consent and if all eligibility criteria are fulfilled. Any candidate taking one of the prohibited medication as listed in the exclusion criteria (see paragraph 7.1) must be willing to start the trial with a 28 day wash out phase in order to start the study treatment phase.

All study participants will receive a flat rate of 200 CHF to compensate for travel cost associated with frequent study site visits.

7.3 Assignment to study groups

All patients will undergo treatment with indapamide, chlorthalidone, and hydrochlorothiazide in a random sequence without stratification. Allocation will be concealed using sequentially coded drug bottles that are otherwise identical. Preparation and handling of the unblinded drug bottles will be done at a facility otherwise not involved in the trial (Apotheke Dr. Hysek AG). Randomization lists will be generated and stored at DCR with no access for persons directly involved in the trial. An independent data manager at DCR will communicate directly with Apotheke Dr. Hysek AG, which prepares the drug bottles.

7.4 Criteria for withdrawal / discontinuation of participants

7.4.1 Criteria for early discontinuation of study participation

Study participants must be withdrawn from the study if the following occurs:

- At the participants’ own request.
- If, in the investigator's opinion, continuation of the study would be harmful to the participant’s well-being.
- If the participant discontinues treatment before the assigned second treatment period is completed.

- Use of prohibited medication indicated during study (see paragraph 8.6.2).
- Severe hypokalemia (plasma potassium <2.5 mmol/L) (see paragraph 9.2.4.2).
- Uric acid lowering therapy (see paragraph 9.2.4.2).
- Pregnancy (see paragraph 10.1.2).

Participants lost to follow-up or withdrawn before two treatment periods are completed will be accounted for by randomizing additional participants to reach a final number of 99 patients completing the study (see also paragraph 11.2). Given the study design (three different active treatment periods, frequent study visits) and the potent thiazides studied, we assume an overall drop-out rate of 20 %. Hence, we expect 124 patients that need to be randomized to reach 99 patients completing the study.

8. STUDY INTERVENTION

8.1 Identity of investigational medicinal products

8.1.1 Experimental intervention

Capsules containing 2.5 mg indapamide or 25 mg chlorthalidone will be supplied by Apotheke Dr. Hysek AG, and patients will take 1 capsule per day in the morning with no dose modifications possible. Patients will be randomly allocated to one of the six treatment sequences. For further details see paragraph 6.1 and Figure 1.

Active treatment periods can be extended by 7 days, but patients must be taking the IMP during the 24-hour urine collection at the end of the treatment period. Wash out periods can be extended to a maximum of 56 days. Duration of active treatment and wash out periods (28 days each) were chosen to ensure stable steady-state plasma concentrations for all three thiazides, of which chlorthalidone has the longest half-life, up to 60 hours with long term administration. For determination of active treatment and wash out duration, we additionally considered the fact that the pharmacodynamic response of thiazides is significantly longer than the predicted by half-life [40].

8.1.2 Control intervention (standard / routine / comparator treatment)

Capsules containing 50 mg hydrochlorothiazide will be supplied by Apotheke Dr. Hysek AG and patients will take 1 capsule per day in the morning with no dose modifications possible, following the same circumstances as already explained under paragraph 8.1.1.

8.1.3 Packaging, labelling and supply (re-supply)

IMP will be provided in identically looking bottles containing identically looking capsules (DB capsules size A) and labeled with trial-specific labels:

- Name, address, and telephone number of the sponsor
- Pharmaceutical dosage form (capsules), route of administration (per os), number of capsules
- Batch number, Lot number
- Trial acronym (INDAPACHLOR Trial)
- Trial subject identification number
- Storage conditions
- Expiry date
- Safety warning “Keep out of reach of children”
- Randomization number

8.1.4 Storage conditions

IMP will be stored in a securely locked cabinet. Access will be limited to investigators and their designees. Neither investigators nor any designees may provide IMP to any subject not participating in this study. The IMP will be stored according to the conditions specified in the IMP label. The temperature in the drug storage area is recorded on a continuous (24/7) basis using an automated recording system.

8.2 Administration of experimental and control interventions

8.2.1 Experimental intervention

Indapamide and chlorthalidone capsules will be taken once daily per os in the morning. For pharmacokinetics see Table 2.

8.2.2 Control intervention

Hydrochlorothiazide capsules will be taken once daily per os in the morning, identical to indapamide and chlorthalidone capsules. For pharmacokinetics of thiazides used in this study see Table 2.

Table 2. *Pharmacokinetics of thiazides (based on [40-44])*

Drug	*Peak, h*	*Half-life, h*	*Metabolism/Route of Elimination*
Hydrochlorothiazide	*4-6*	*6-15*	*100 % kidney (unchanged)*
Chlorthalidone	*2-6*	*45-60*	*60 % kidney (unchanged), 40 % liver*
Indapamide	*2-3*	*14-18*	*>90 % liver (metabolized by CYP3A4)*

8.3 Dose modifications

No dose modifications are foreseen.

8.4 Compliance with study intervention

Due to the relatively short study time, we do not anticipate major problems with study intervention compliance. Nevertheless, at initial IMP dispensing and the follow-up visits, patients will be reminded about study guidelines, instructed on IMP intake and storage, the 24-hour urine collection procedure, and reminded to arrive at study visits with at least 6 hours fasting. Further, we will instruct patients to call the clinic if they are experiencing problems possibly related to the study product or lost IMP. To enhance validity of data, medication adherence will be assessed by pill counts. Participants will return the unused capsules and bottles at each follow-up visit.

8.5 Data / sample collection and follow-up for withdrawn participants

Participants who are withdrawn from further study participation will be contacted for the EOS safety follow-up assessment 30 days after study withdrawal or treatment discontinuation respectively as specified in the study schedule.

If a participant withdraws consent from the study, the samples collected until the time point of withdrawal will still be analysed and all collected data will be included in the analysis of the study. As it will not be possible to anonymize data, the data will remain coded in the study database.

If a participant withdraws consent for storage of blood and urine samples in the biobank, these samples will be destroyed.

8.6 Trial specific preventive measures

8.6.1 Dietary guidelines

Dietary interventions form the basis of stone prevention. As such, any trial assessing pharmacologic interventions for the prevention of stone recurrence must compare the added efficacy of drug treatment in the setting of non-pharmacologic interventions according to current guidelines [34-36]. At baseline and follow-up visits, we will give patients individualized dietary advice tailored to their 24-hour urine results. To this end, we will emphasize circadian fluid intake to ensure daily urinary volumes of at least 2.5 L/day, a balanced diet rich in fruits, vegetables, and fibers with normal calcium content (1-1.2 g/day) but limited NaCl (<6 g/day) and animal protein (0.8 – 1 g/kg body weight/day) content.

8.6.2 Prohibited medication

The following concomitant medication will not be allowed during study participation:

- thiazide or loop diuretics
- carbonic anhydrase inhibitors (including topiramate)
- xanthine oxidase inhibitors
- alkali
- active vitamin D (calcitriol or similar)
- calcium supplementation
- bisphosphonates
- denusomab
- teriparatide
- sodium-glucose co-transporter 2 (SGLT2) inhibitors
- strong CYP3A4 inhibitors or inducers (may affect indapamide metabolism)

– lithium

If treatment with one of the drugs listed above is indicated during participation in the study, the study participant must be excluded from further participation.

8.6.3 Contraception

Women of child-bearing potential (defined as women who are not surgically sterilized/hysterectomized, and/or who are postmenopausal for less than 12 months), must use an effective contraception method during treatment with IMP. Acceptable methods include:

- hormonal contraception to inhibit ovulation (tablet [“pill”/”mini-pill"], injection, implant, patch, or vaginal ring)
- hormonal intrauterine device
- copper intrauterine device (coil, chain, ball)
- bilateral tubal occlusion
- vasectomized partner (provided that partner is the sole sexual partner of trial participant and that the vasectomized partner has received medical assessment of the surgical success)

Urine pregnancy tests must be performed prior to the start of each treatment period.

8.6.4 Procedures following Adverse Events

Hypokalemia

Hypokalemia (defined as plasma potassium <3.5 mmol/L) is a common side effect of thiazides and is causally linked to the development of hypocitraturia. Hence, we will ensure that all patients remain normokalemic during the trial (plasma potassium 3.5-4.5 mmol/L). For this, a blood draw will be performed in the second week of each treatment period. In the case of mild hypokalemia (3.0-3.4 mmol/L), supplementation with 40 mmol oral potassium chloride (KCL RETARD Hausmann®; 10 mmol potassium chloride per tablet) daily will be started. In the case of moderate hypokalemia (2.5-2.9 mmol/L), supplementation with 80 mmol oral KCl daily will be started. In case of severe hypokalemia (plasma potassium <2.5 mmol/L), the patient will be withdrawn from the study. All cases of hypokalemia will be reported as adverse events.

Gout

Thiazides increase serum uric acid levels which can cause gout. An acute gout flare should be treated symptomatically with analgesics. Xanthine oxidase inhibitors to lower serum and urine uric acid levels can affect urine RSRs and are therefore not allowed during the trial. If a uric acid lowering therapy is needed, the patient will be withdrawn from the study and this will be reported as an adverse event.

HbA1c

Thiazides may worsen glucose tolerance or induce diabetes mellitus. If overt diabetes mellitus develops during the trial (defined as HbA1c ≥6.5 %), the patient will be referred to a diabetologist. New onset diabetes mellitus will be reported as adverse event. SGLT2 inhibitors are not allowed for treatment because they may influence urine supersaturations [45, 46].

8.7 Concomitant interventions (treatments)

All medications not listed in paragraph 8.6.2 will be allowed before and during the study as concomitant treatments. The use of concomitant medications will be recorded in the electronic Case Report Form (eCRF). There are no restrictions to medications or treatments after the study. All urological interventions related to symptomatic and asymptomatic stone disease are permitted during the trial. Interventional treatment of asymptomatic stones of patients enrolled in the trial will be left to the discretion of the treating urologists.

8.8 Study drug accountability

An inventory record of IMP will be maintained at the site level (IMP received from the supplier, unused IMP destroyed on site) and at the patient level (dispensed to the patient, returned by the patient).

8.9 Return or destruction of study drug

Upon completion or termination of the trial, all unused IMP will be destroyed following standard procedures for medication destruction and the destruction documented. Documentation on IMP

destruction will be provided in the Trial Master File.

9. STUDY ASSESSMENTS

9.1 Study flow chart(s) / table of study procedures and assessments

For the schedule of assessments refer to “Study Schedule” table at the beginning of this document.

9.2 Assessments of outcomes

9.2.1 Assessment of primary outcome

Patients will collect 24-hour urines at the beginning (the day before IMP start) and at the end of each treatment period (last day of IMP intake) to measure urine parameters.

9.2.2 Assessment of secondary outcomes

Blood samples will be taken in fasting condition (at least 6 hours) at the beginning (the first day of IMP intake, before IMP intake) and at the end of each treatment period (last day of IMP intake). Systolic and diastolic blood pressure, heart rate, height (measured only once at the screening or baseline visit), and body weight will also be recorded at these time points.

9.2.3 Assessment of other outcomes of interest

Fasting second morning urines will be collected at the beginning (the first day of IMP intake, before IMP intake, but after the end of the 24-hour urine collection) and at the end of each treatment period (on the last day of IMP intake, after the end of the 24-hour urine collection) for isolation of urinary extracellular vesicles (uEVs).

At the beginning (the first day of IMP intake, before IMP intake) and at the end of each treatment period, blood (at least 6 hours fasting) and an aliquot of the 24-hour urine will be collected for the INDAPACHLOR Biobank. This material will be used for future biomedical projects.

9.2.4 Assessment of safety outcomes

9.2.4.1 Serious adverse events (SAEs)

SAEs will be collected, fully investigated, and documented in the source documents and the eCRF for all participants from the date of signature of the informed consent form until the last protocol-specific procedure has been completed, including a safety follow-up period of 30 days after end of study treatment.

9.2.4.2 Adverse Events of Special Interest (AESIs)

Adverse events of special interest (AESIs) are AEs that may occur during IMP intake. The following events are considered as AESIs:

- Hypokalemia (defined as plasma potassium <3.5 mmol/L)
- Uric acid levels that need a uric acid lowering therapy
- New onset diabetes mellitus
- AEs that require discontinuation of IMP treatment:
  - Use of prohibited medication indicated during study (see paragraph 8.6.2).
  - Severe hypokalemia (plasma potassium <2.5 mmol/L) (see paragraph 9.2.4.2).
  - Uric acid lowering therapy (see paragraph 9.2.4.2).
  - Pregnancy (see paragraph 10.1.2).

9.2.4.3 Vital signs

Assessment of vital signs (heart rate, systolic and diastolic blood pressure at the right arm after at least 5 minutes at rest) will be performed with a calibrated automated sphygmomanometer in a seated position after at least 5 minutes of rest.

9.2.5 Assessments in participants who prematurely stop the study

See paragraph 8.5.

9.3 Procedures at each visit

9.3.1 Visit or phone call 1 (screening day 1*; - 30 days to -15 days)

- Patient information and delivery of written study information to subject
- Pre-assessment of eligibility (based on data available per routine)
- Demographics
- Medical history
- Stone composition (all stones analyzed prior to randomization as available)
- General physical examination, including body weight, height
- Vital signs (heart rate, systolic and diastolic blood pressure at the right arm after at least 5 minutes at rest in a sitting position)
- Concomitant medication

* For participants in current follow-up for kidney stone disease, all assessments of visit 1 and visit 2 are performed at visit 3. For participants with first time work-up for kidney stone disease (new referrals), screening assessments should be performed at visits 1 and 2, unless the distribution over 2 visits is impracticable, in which case the same procedure as for participants in current follow-up applies.

9.3.2 Visit 2 (screening day 2; -14 days to day 0)

- Written informed consent (if not obtained earlier). Note: In order to give subjects in current follow-up for past stone disease sufficient time for the decision on study participation, initial patient information may be done via phone and the written study information provided to the subject via postal or electronic mail prior to the visit; same for new referrals if they are unable to come extra for visit 1
- Assessment of eligibility
- Blood analysis (after at least 6 hours fasting)*: Sodium, potassium, chloride, calcium, magnesium, phosphate, creatinine, urea, uric acid, venous blood gas, PTH, 25-OH Vitamin D, 1,25-OH Vitamin D, glucose, HbA1c
- Urine analysis (one 24-hour urine collection)*: Sodium, potassium, chloride, calcium, magnesium, phosphate, creatinine, urea, uric acid, oxalate, citrate, sulfate, ammonium, bicarbonate, pCO_2, pH, volume
- SAEs and AEs related to SAEs
- AESIs (see paragraph 9.2.4.2)
- Concomitant medication

* Blood and urine analyses performed are not study-specific but part of the clinical routine in the work-up or follow-up of kidney stone formers.

9.3.3 Between Visit 2 and Visit 3 (day 0 to day 1)

24 hours before visit 3, the patient starts collecting a 24-hour urine that will be taken to the visit for laboratory analysis. The day before the collection is supposed to start, a reminder will be sent to the participant by SMS. If Visit 1 and Visit 2 merge under the conditions described in paragraph 9.3.1, this paragraph doesn't apply.

9.3.4 Visit 3 (day 1) – Baseline visit

- Re-assessment of eligibility
- Concomitant medication
- Pregnancy test (from urine) for women of child-bearing potential (defined as women who are not surgically sterilized/hysterectomized, and/or who are postmenopausal for less than 12 months). Women of child-bearing potential must use an effective contraceptive during treatment with IMP, such as oral contraceptives or intrauterine devices
- Randomization
- Vital signs (heart rate, systolic and diastolic blood pressure at the right arm after at least 5 minutes at rest in a sitting position)
- General physical examination, including body weight and height (if not measured at screening)
- Blood analysis (after at least 6 hours fasting). For details regarding the parameters assessed, see

paragraph 9.3.2 (Visit 2).
- Urine analysis (uEVs, after at least 6 hours fasting). For details regarding the parameters assessed, see paragraph 9.3.2 (Visit 2).
- Collection of biobank material: 7.5 mL of EDTA plasma, 7.5 mL of serum, 20 mL of 24 h urine, 7.5 mL of EDTA blood for DNA extraction
- SAEs and AEs related to SAEs
- AESIs (see paragraph 9.2.4.2)
- Instructions on non-pharmacologic recommendations for stone prevention
- Hand out of first IMP
- Handout of canister for end of first treatment phase 24h-hour urine collection

Daily intake of IMP starts the day of visit 3 (day 1) and continues until day +28. If the patient is not able to come to visit 5 at day +28, an extension of up to 7 days can be allowed (the patient must keep taking medication during this time).

9.3.5 Visit 4 (7-14 days after Visit 3)

7-14 days after visit 3, a plasma potassium will be measured (see paragraph 8.6.4) and AEs of special interest, SAEs and AEs related to SAEs will be assessed.

9.3.6 Between Visit 4 and Visit 5 (24h before Visit 5)

24 hours before visit 5, the patient starts collecting a 24-hour urine that will be taken to the visit for laboratory analysis. The day before the collection is supposed to start, a reminder will be sent to the participant by SMS.

9.3.7 Visit 5 (day +28 + up to 7 days)

- Vital signs (heart rate, systolic and diastolic blood pressure at the right arm after at least 5 minutes at rest in a sitting position)
- General physical examination, including body weight
- Concomitant medication
- Blood analysis (after at least 6 hours fasting). For details regarding the parameters assessed, see paragraph 9.3.2 (Visit 2).
- Urine analysis (uEVs, after at least 6 hours fasting). For details regarding the parameters assessed, see paragraph 9.3.2 (Visit 2).
- Collection of biobank material: 7.5 mL of EDTA plasma, 7.5 mL of serum, 20 mL of 24 h urine.
- SAEs and AEs related to SAEs
- AESIs (see paragraph 9.2.4.2)
- Collection of used IMP bottle
- Handout of canister for baseline 24h-hour urine collection of second treatment phase

Visit 5 is followed by a 28 day wash out, which starts on day +28 and continues until day +56 (assuming a wash out phase of 28 days; the numbers may vary according to the effective length of the wash out phase which can be maximally 56 days).

9.3.8 Between Visit 5 and Visit 6 (24h before Visit 6)

24 hours before visit 5, the patient starts collecting a 24-hour urine that will be taken to the visit for laboratory analysis. The day before the collection is supposed to start, a reminder will be sent to the participant by SMS.

9.3.9 Visit 6 (day +57)

- Vital signs (heart rate, systolic and diastolic blood pressure at the right arm after at least 5 minutes at rest in a sitting position)
- General physical examination, including body weight
- Concomitant medication
- Pregnancy test (from urine) for women of child-bearing potential (defined as women who are not surgically sterilized/hysterectomized, and/or who are postmenopausal for less than 12 months). Women of child-bearing potential must use an effective contraceptive during treatment with IMP, such as oral contraceptives or intrauterine devices
- Blood analysis (after at least 6 hours fasting). For details regarding the parameters assessed, see

paragraph 9.3.2 (Visit 2).
- Urine analysis (uEVs, after at least 6 hours fasting). For details regarding the parameters assessed, see paragraph 9.3.2 (Visit 2).
- Collection of biobank material: 7.5 mL of EDTA plasma, 7.5 mL of serum, 20 mL of 24 h urine.
- SAEs and AEs related to SAEs
- AESIs (see paragraph 9.2.4.2)
- Instructions on non-pharmacologic recommendations for stone prevention
- Hand out of second IMP
- Handout of canister for end of second treatment phase 24h-hour urine collection

Daily intake of IMP starts the day of visit 6 (day +57) and continues until day +84. If the patient is not able to come to visit 8 at day +84, an extension of up to 7 days can be allowed (the patient must keep taking medication during this time).

9.3.10 Visit 7 (7-14 days after Visit 6)

7-14 days after visit 6, a plasma potassium will be measured (see paragraph 8.6.4) and AEs of special interest, SAEs and AEs related to SAEs will be assessed.

9.3.11 Between Visit 7 and Visit 8 (24h before Visit 8)

24 hours before visit 6 the patient starts collecting a 24-hour urine that will be taken to the visit for laboratory analysis. The day before the collection is supposed to start, a reminder will be sent to the participant by SMS.

9.3.12 Visit 8 (day +84 + up to 7 days)

- Vital signs (heart rate, systolic and diastolic blood pressure at the right arm after at least 5 minutes at rest in a sitting position)
- General physical examination, including body weight
- Concomitant medication
- Blood analysis (after at least 6 hours fasting). For details regarding the parameters assessed, see paragraph 9.3.2 (Visit 2).
- Urine analysis (uEVs, after at least 6 hours fasting). For details regarding the parameters assessed, see paragraph 9.3.2 (Visit 2).
- Collection of biobank material: 7.5 mL of EDTA plasma, 7.5 mL of serum, 20 mL of 24 h urine.
- SAEs and AEs related to SAEs
- AESIs (see paragraph 9.2.4.2)
- Collection of used IMP bottle
- Handout of canister for baseline 24h-hour urine collection of third treatment phase

Visit 8 is followed by a 28 day wash out, which starts on day +84 and continues until day +112 (assuming a wash out phase of 28 days; the numbers may vary according to the effective length of the wash out phase which can be maximally 56 days).

9.3.13 Between Visit 8 and Visit 9 (24h before Visit 9)

24 hours before Visit 9, the patient starts collecting a 24-hour urine that will be taken to the visit for laboratory analysis. The day before the collection is supposed to start, a reminder will be sent to the participant by SMS.

9.3.14 Visit 9 (day +113)

- Vital signs (heart rate, systolic and diastolic blood pressure at the right arm after at least 5 minutes at rest in a sitting position)
- General physical examination, including body weight
- Concomitant medication
- Pregnancy test (from urine) for women of child-bearing potential (defined as women who are not surgically sterilized/hysterectomized, and/or who are postmenopausal for less than 12 months). Women of child-bearing potential must use an effective contraceptive during treatment with IMP, such as oral contraceptives or intrauterine devices
- Blood analysis (after at least 6 hours fasting). For details regarding the parameters assessed, see paragraph 9.3.2 (Visit 2).

- Urine analysis (uEVs, after at least 6 hours fasting). For details regarding the parameters assessed, see paragraph 9.3.2 (Visit 2).
- Collection of biobank material: 7.5 mL of EDTA plasma, 7.5 mL of serum, 20 mL of 24 h urine.
- SAEs and AEs related to SAEs
- AESIs (see paragraph 9.2.4.2)
- Instructions on non-pharmacologic recommendations for stone prevention
- Hand out of third IMP
- Handout of canister for end of third treatment phase 24h-hour urine collection

Daily intake of IMP starts the day of visit 9 (day +113) and continues until day +140. If the patient is not able to come to visit 11 at day +140, an extension of up to 7 days can be allowed (the patient must keep taking medication during this time).

9.3.15 Visit 10 (7-14 days after Visit 9)

7-14 days after visit 9, a plasma potassium will be measured (see paragraph 8.6.4) and AEs of special interest, SAEs and AEs related to SAEs will be assessed.

9.3.16 Between Visit 10 and Visit 11 (24h before Visit 11)

724 hours before visit 11, the patient starts collecting a 24-hour urine that will be taken to the visit for laboratory analysis. The day before the collection is supposed to start, a reminder will be sent to the participant by SMS.

9.3.17 Visit 11 (day +140 + up to 7 days)

- Vital signs (heart rate, systolic and diastolic blood pressure at the right arm after at least 5 minutes at rest in a sitting position)
- General physical examination, including body weight
- Concomitant medication
- Blood analysis (after at least 6 hours fasting). For details regarding the parameters assessed, see paragraph 9.3.2 (Visit 2).
- Urine analysis (uEVs, after at least 6 hours fasting). For details regarding the parameters assessed, see paragraph 9.3.2 (Visit 2).
- Collection of biobank material: 7.5 mL of EDTA plasma, 7.5 mL of serum, 20 mL of 24 h urine.
- SAEs and AEs related to SAEs
- AESIs (see paragraph 9.2.4.2)
- Collection of used IMP bottle

9.3.18 Safety Follow-up (phone call; 30 days ±7 days after Visit 11)

- SAEs and AEs related to SAEs
- AESIs (see paragraph 9.2.4.2)

10. SAFETY

10.1 Drug studies

During the entire duration of the study, the following events are collected, fully investigated, and documented in source documents and in the eCRF:

- Serious Adverse Events (SAEs)
- AEs relevant to a reported SAE
- AESIs (see paragraph 9.2.4.2)

Study duration encompasses the time from when the participant signs the informed consent form until the last protocol-specific procedure has been completed, including the safety follow-up visit 30 days after end of treatment (end of study visit).

10.1.1 Definition and assessment of (serious) adverse events and other safety related events

An **Adverse Event (AE)** is any untoward medical occurrence in a patient or a clinical investigation participant administered a pharmaceutical product and which does not necessarily have a causal

relationship with the study procedure. An AE can therefore be any unfavourable and unintended sign (including an abnormal laboratory finding), symptom, or disease temporally associated with the use of a medicinal (investigational) product, whether or not related to the medicinal (investigational) product. [ICH E6 1.2]

A **Serious Adverse Event (SAE)** is classified as any untoward medical occurrence that:

- results in death,
- is life-threatening,
- requires in-patient hospitalization or prolongation of existing hospitalisation,
- results in persistent or significant disability/incapacity, or
- results in a congenital anomaly/birth defect.

In addition, important medical events that may not be immediately life-threatening or result in death, or require hospitalisation, but may jeopardise the patient or may require intervention to prevent one of the other outcomes listed above should also usually be considered serious. [ICH-E2A]

SAEs should be followed until resolution or stabilisation. Participants with ongoing SAEs at study termination (including safety follow-up visit) will be further followed up until recovery or until stabilisation of the disease after termination.

Patients may be hospitalized for administrative or social reasons during the study. These and other hospitalizations planned at the beginning of the study do not need to be reported as a SAE in case they have been reported at screening visit in the source data and have been performed as planned.

Severity of SAEs
The assessment of SAEs' severity will be performed in accordance with the "Common Terminology Criteria for Adverse Events" (CTCAE) Version 5.0.

Assessment of causality

Medical judgment should be used to determine the relationship, considering all relevant factors, including pattern of reaction, temporal relationship, de-challenge or re-challenge, confounding factors such as concomitant medication, concomitant diseases and relevant history. Assessment of causal relationship should be recorded in the eCRF. The eCRF will allow assessment of causality for all three potential IMPs to maintain blinding.

Yes: There is a reasonable causal relationship between the IMP administered and the AE.
No: There is no reasonable causal relationship between the IMP administered and the AE.

Worsening of the underlying disease or other pre-existing conditions

Worsening of the underlying disease or of other pre-existing conditions will be recorded as an (S)AE in the eCRF provided the event is a reportable event as defined under paragraph 10.1.

Unexpected Adverse Drug Reaction

An "unexpected" adverse drug reaction is an adverse reaction, the nature or severity of which is not consistent with the applicable product information (Product Information for approved drugs hydrochlorothiazide, indapamide and chlorthalidone). [ICH-E2A]

Suspected Unexpected Serious Adverse Reactions (SUSARs)

The sponsor evaluates any SAE that has been reported regarding seriousness and causality. To maintain blinding, the initial assessment will be done assuming that any of the three IMPs is the underlying treatment. If the event is judged to be potentially related to at least one of the IMPs, the event is forwarded to Apotheke Dr. Hysek AG for unblinding and possibly further processing. If the event is actually unexpected, it is classified as a SUSAR and the sponsor is notified accordingly.

10.1.2 Reporting of serious adverse events (SAE) and other safety related events

Reporting of SAEs

All SAEs must be reported immediately and within a maximum of 24 hours of learning of its occurrence to the sponsor via the SAE report form in secuTrial. The sponsor will re-evaluate the SAE in secuTrial.

AEs that require discontinuation of IMP

AEs that require discontinuation of IMP treatment (defined as AESIs, see section 9.2.4.2) should be reported to the sponsor within 3 days.

Reporting of SUSARs

The sponsor reports a SUSAR to the CEC and to Swissmedic within 7 days, if the event is life-threatening or fatal, or within 15 days (all other events).

Reporting of safety signals

All suspected new risks and relevant new aspects of known adverse reactions that require safety-related measures, i.e. so called safety signals, must be reported to the sponsor within 24 hours. The sponsor reports the safety signals within 7 days to the CEC and to Swissmedic. The sponsor must immediately inform all participating investigators about all safety signals.

Reporting and handling of pregnancies

Pregnant participants must immediately discontinue IMP and will be excluded from further study participation (see paragraph 7.4.1). Any pregnancy during the treatment phase of the study and within 30 days after discontinuation of IMP must be reported to the sponsor via the SAE reporting form in the eCRF within 24 hours. The course and outcome of the pregnancy will be followed up carefully and any abnormal outcome regarding the mother or the child should be documented and reported.

Periodic reporting of safety and general progress of the clinical trial

An annual safety report / general study progress report will be prepared and submitted once a year to the local CEC and to Swissmedic by the sponsor. The report is written in compliance to the ICH-E2F guideline taking into account the requirements for non-commercial sponsors. The start date for the report is the date of the sponsor’s first authorisation to conduct the trial. The report is submitted to the CEC and to Swissmedic throughout the duration of the trial, and the last submission of the report will cover the Last Participant Last Visit.

10.1.3 Follow up of (Serious) Adverse Events

Participants with any reported ongoing SAE or AESIs (as defined in paragraph 9.2.4.2) at the last scheduled study contact will be followed until resolution of the event or a stabilized condition of the subject has been achieved or until the subject is lost to follow-up.

Any new SAEs the investigator gets aware of that occur after the last scheduled study contact and are determined by the investigator to be reasonably associated with the use of the IMP, should be reported to the sponsor. The investigator should follow potentially IMP-related SAEs identified after the last scheduled contact (and report any significant follow-up information to the sponsor) until the events are resolved or stabilized, or the subject is lost to follow-up.

11. STATISTICAL METHODS

11.1 Hypothesis

The statistical null hypothesis that there is no difference in change in urinary supersaturations with treatment of indapamide or chlorthalidone compared to hydrochlorothiazide will be tested against the alternative that indapamide or chlorthalidone are superior to hydrochlorothiazide in reducing at least one of the two urine supersaturations (RSR CaOx and RSR CaP).

11.2 Determination of sample size

The sample size calculation was based on the following assumptions:

- Allocation ratio: 1:1:1 (3-period crossover design)
- Type I and II error rate: 0.1 (two-sided) and 0.2, respectively
- Effect measure: Difference in change of urine RSR CaOx and RSR CaP
- Analysis approach: Repeated-measures model adjusted for baseline
- Correction for dropouts: Patients lost or withdrawn before two treatment periods are completed will be accounted for by randomizing additional patients

– Correction for multiple testing: Due to the exploratory nature of the study, no adjustment for multiple testing is done
– Expected mean values, standard deviations, and correlations of RSRs CaOx and CaP were calculated from NOSTONE data [21], which had identical eligibility criteria compared to INDAPACHLOR:
    - Assumptions for CaOx RSR
        - Expected mean value: 7.5
        - Expected standard deviation: 5
        - Expected difference: 20 % (i.e. -1.5 in absolute terms)
        - Expected correlation: 0.4
    - Assumptions for CaP RSR
        - Expected mean value: 2.5
        - Expected standard deviation: 2.3
        - Expected difference: 20 % (i.e. -0.5 in absolute terms)
        - Expected correlation: 0.5

The expected difference of 20 % RSR reduction with indapamide or chlorthalidone compared to hydrochlorothiazide was chosen because this would translate into a clinically meaningful reduction of recurrence risk. In a past dietary RCT of 5 year duration, a 20 % reduction of RSR CaOx at 1 week compared to baseline was associated with a 16 % reduction in recurrence (a composite of symptomatic or radiologic recurrence) during follow-up [47]. Our (unpublished) post-hoc analysis of NOSTONE revealed similar results: a 20 % reduction of RSR CaOx at 3 months compared to baseline was associated with a 15 % reduction in recurrence (a composite of symptomatic or radiologic recurrence) during follow-up. RSR CaOx and CaP were also significantly associated with radiologic recurrence on CT in NOSTONE, the most sensitive recurrence parameter. A 20 % lower baseline value of RSR CaOx or RSR CaP was associated with a 7.5 %, respectively 9 % lower risk of radiologic recurrence during follow-up. Based on the methods and assumptions described above, the resulting total sample size for RSR CaOx and RSR CaP is 58 and 99 patients, respectively. To ensure sufficient power for both primary outcomes, the larger sample size is chosen for this trial. Given the study design (three different active treatment periods, frequent study visits) and the potent thiazides studied, we assume an overall drop-out rate of 20 %. Hence, we expect 124 patients that need to be randomized to reach 99 patients completing the study

11.3 Statistical criteria of termination of trial

Not applicable.

11.4 Planned analyses

The statistical analysis of the trial will be done at DCR by a statistician blinded to the allocated sequence. This process is defined in standard operating procedures. Before 50 % of participants are enrolled, a detailed statistical analysis plan will be written. The plan will determine all necessary data preparation steps (e.g. additional validations, generation of new variables), definitions (e.g. analysis sets), and statistical analyses (e.g. models, outputs such as tables and graphs).

11.4.1 Datasets to be analysed, analysis populations

The primary analysis will include all randomized patients that completed at least two treatment periods. In a per-protocol analysis, only patients that completed all three periods and had no major protocol deviations will be considered.

11.4.2 Primary analysis

Regarding the two primary outcomes RSR CaOx and RSR CaP, indapamide and chlorthalidone will be compared to hydrochlorothiazide. Due to the exploratory nature of the study, no adjustment for multiple testing will be done. In a context where promising findings will undergo subsequent outcome trial validation, the necessity for multiple-testing correction diminishes, as false positive results will not alter practice [48]. Consequently, we will use a type-I error rate for individual hypothesis tests at a two-sided level of 0.1, as this approach aligns with our objectives and maintains rigor while allowing for potential discoveries. To investigate the effect of indapamide and chlorthalidone on the primary outcomes (RSR

CaOx and RSR CaP), we will use linear mixed effects models using the log-transformed outcome as dependent variable, fixed effects for the respective log-transformed baseline value, indicators of treatment (categorical) and period (categorical), and a random intercept for the patient. From this model, we will estimate the relative difference (ratio) between pairwise treatments (indapamide vs hydrochlorothiazide and chlorthalidone vs hydrochlorothiazide) and the associated 95 % confidence interval. Moreover, we will also calculate the relative change (ratio) from baseline to week 4 within each treatment.

11.4.3 Secondary analyses

Secondary continuous outcomes will be analysed likewise and values log-transformed if required.

11.4.4 Interim analyses

No interim analysis is foreseen.

11.4.5 Safety analysis

Safety outcomes will be presented in a descriptive way.

11.4.5.1 Exploratory analysis

Total and phosphorylated NCC will be detected and quantified on immunoblots with highly sensitive and specific antibodies, normalized to the uEV housekeeping protein Alix [49, 50]. To ensure validity of results, we will perform an independent normalization to a second uEV housekeeping protein, CD9 [51]. Changes from baseline in uEV NCC abundance induced by the three thiazides will be compared using an ordinary linear regression model adjusted for treatment-period, after log-transformation of the dependent variables. Further, in a pooled analysis ignoring treatment type, an analysis of the strength and direction of the correlation between changes in uEV NCC abundance and changes in urine calcium, urine citrate, urine RSR CaOx and RSR CaP and plasma potassium will be initiated. The correlation analysis will be conducted using Pearson's or Spearman's correlation coefficient, depending on data distribution.

11.4.6 Deviation(s) from the original statistical plan

Deviations from the statistical analysis plan will be stated and justified in the final analysis report.

11.5 Handling of missing data and drop-outs

Drop-outs will be censored at the last available visit. No data imputation will be performed in case of missing information.

12. QUALITY ASSURANCE AND CONTROL

12.1 Data handling and record keeping/archiving

12.1.1 Case report forms

The investigators will maintain appropriate medical and research records for this trial, in compliance with ICH-E6 (GCP) and regulatory and institutional requirements for the protection of confidentiality of subjects. The investigators and any other representatives of the study team will have access to the records as needed. The principal investigator will permit authorized representatives of the sponsor and regulatory agencies to examine clinical records for the purposes of quality assurance reviews, audits, and evaluation of the study safety and progress.

The CRF will be electronic. All data requested on the eCRF must be recorded and the recorded data should be consistent with the source documents or the discrepancies should be explained. The investigator should ensure the accuracy, completeness, and timeliness of the data reported in the eCRF and all other required reports. Generally, the eCRF should be completed within two weeks of completion of a participant's visit. Authorized to enter data into the eCRF are the local trial team staff. The principal investigator is responsible for proper training and instruction of the trial personnel filling data into the eCRF. Study-related data of the patient will be collected in a coded manner. The names of the patients will not be disclosed. A code (unique, consecutive numbered) will be attributed to each patient registered: 001, 002, etc.).

12.1.2 Specification of source documents

Source documents must be available at the site to document the existence of the study participants and must include the original documents relating to the study, as well as the medical treatment and medical history of the participant.

Any change or correction to source data should be dated, initialled, and explained (if necessary) and should not obscure the original entry.

All data captured in the eCRF should be itemised on a source data location list, which will be stored in the Investigator Site File at each study site. This list should clearly indicate the source data location corresponding to each eCRF entry. If several sources are possible for one eCRF entry, the priority order of these must be specified in the list. There are no study data that will be directly entered into the eCRF, i.e., the eCRF will not be the source for any data points.

12.1.3 Record keeping/archiving

All study data (written and electronic), must be retained for a period of at least 20 years from the completion or premature termination of the trial. The investigator should take measures to prevent accidental or premature destruction of these documents.

12.2 Data management

12.2.1 Data management system

The CRFs in this trial are implemented electronically using a dedicated Electronic Data Capture (EDC) system (secuTrial). The EDC system is activated for the trial only after successfully passing a formal test procedure. All data entered in the eCRF are stored on a Linux server in a dedicated Oracle database.

Responsibility for hosting the EDC system and the database lies with Inselspital Bern.

12.2.2 Data security, access and back-up

The server hosting the EDC system and the database is kept in a locked server-room. Only the system administrators have direct access to the server. A role concept with personal passwords (site investigator, statistician, monitor, administrator, etc.) regulates permission for each user to use the system and database as they require.

All data entered into the eCRF are transferred to the database using Transport Layer Security (TLS) encryption. Each data point has attributes attached to it identifying the user who entered it with the exact time and date. Retrospective alterations of data in the database are recorded in an audit table: Time, table, data field, altered value, and the person are recorded (audit trail). A multi-level back-up system is implemented.

12.2.3 Analysis and archiving

At final analysis, data files will be extracted from the database into statistical packages to be analyzed. After database lock, the status of the database at this time is recorded in special archive tables. The sponsor will keep the Trial Master File, the extracted data, the meta data and final reports for at least 20 years.

12.2.4 Electronic and central data validation

Data is checked by the EDC system for completeness and plausibility. Furthermore, selected data points are cross-checked for plausibility with previously entered data for that participant. In addition, central data reviews will be performed on a regular basis to ensure completeness of the data collected and accuracy of the primary outcome data. Before database lock, the principal investigator will validate the collected data with his signature.

12.3 Monitoring

For quality control of the study conduct and data retrieval, the clinical site will be visited by appropriately trained and qualified monitors. Any findings and comments will be documented in site visit reports and communicated to the sponsor as applicable. Prior to study start (first participant enrolled) a plan detailing all monitoring-related procedures will be developed. All source data and relevant documents will be accessible to monitors and questions of monitors are answered during site visits.

12.4 Audits and inspections

Source data/documents must be available to audits by the sponsor or designee or to inspections by the CA (Swissmedic) or CEC during the study or after its completion. The investigator will support the inspectors in their activities and will answer questions from inspectors as needed. All involved parties must keep the participant data strictly confidential.

12.5 Confidentiality, data protection

The investigator ensures anonymity of the participants; participants will not be identified by names in any study documents leaving the study site. Subject confidentiality will be ensured by utilizing unique subject identification codes (see paragraph 12.1.1). Signed informed consent forms and the patient enrollment log will be kept strictly confidential to enable patient identification at the site. The code list with the patient's identities will be kept at the site in a key-locked room under responsibility of the principal investigator, and only authorized personnel (study site personnel involved in the trial, clinical monitors and competent authorities) will have access to it.

12.6 Storage of biological material and related health data

Plasma, serum, urine, and DNA collected from patients will be stored indefinitely in the INDAPACHLOR Biobank at the Bern University Hospital. The samples will not be destroyed at the end of the study. Patient's samples will be stored only after a separate informed consent is signed. The procedures and rules of the INDAPACHLOR Biobank are described in a specific booklet (Reglement der Biobank für die INDAPACHLOR Studie).

13. PUBLICATION AND DISSEMINATION POLICY

The sponsor will enter and publish a summary of the trial results in a public register in accordance with ClinO Art. 65a within one year of completion or premature termination of the trial. An interruption lasting more than two years is considered a premature termination of the trial.

For the purpose of publication in the public register, the sponsor also ensures that a lay summary of the trial results is entered in BASEC within one year of completion or premature termination of the trial. The entry is made at least in the national languages of Switzerland in which the study participants were recruited.

The investigator will directly provide each study participants with the lay summary of the trial results at the end of the study.

Study results will be presented at national and international meetings and will be submitted for publication to high impact, peer reviewed journals. The submitted manuscript for the primary publication will contain at least results of the primary analysis of all primary and secondary endpoints as defined above. Authorship will be determined based on contribution and criteria of the International Committee of Medical Journal Editors.

Once results have been published, trial data will be accessible to external researchers and coded datasets corresponding to each publication will be made available. Researchers wishing to replicate the analyses or to do an individual patient meta-analysis may request the data from the sponsor.

14. FUNDING AND SUPPORT

14.1 Funding

The trial is financed by a research grant of the medical faculty of the University of Bern (SF Board Call 2023). The trial will also receive intramural support of the Inselspital, Bern University Hospital.

15. INSURANCE

Insurance will be provided by the sponsor. A copy of the certificate will be filed in the Trial Master File.

16. REFERENCES

1. Werness PG, Brown CM, Smith LH, et al. EQUIL2: a BASIC computer program for the calculation of urinary saturation. *J Urol.* 1985;134:1242-1244.
2. Chewcharat A, Curhan G. Trends in the prevalence of kidney stones in the United States from 2007 to 2016. *Urolithiasis.* 2021;49:27-39.
3. Romero V, Akpinar H, Assimos DG. Kidney stones: a global picture of prevalence, incidence, and associated risk factors. *Rev Urol.* 2010;12:e86-96.
4. Scales CD, Jr., Smith AC, Hanley JM, et al. Prevalence of kidney stones in the United States. *Eur Urol.* 2012;62:160-165.
5. Ferraro PM, Curhan GC, D'Addessi A, et al. Risk of recurrence of idiopathic calcium kidney stones: analysis of data from the literature. *J Nephrol.* 2017;30:227-233.
6. New F, Somani BK. A Complete World Literature Review of Quality of Life (QOL) in Patients with Kidney Stone Disease (KSD). *Curr Urol Rep.* 2016;17:88.
7. Saigal CS, Joyce G, Timilsina AR. Direct and indirect costs of nephrolithiasis in an employed population: opportunity for disease management? *Kidney Int.* 2005;68:1808-1814.
8. Lotan Y, Cadeddu JA, Roerhborn CG, et al. Cost-effectiveness of medical management strategies for nephrolithiasis. *J Urol.* 2004;172:2275-2281.
9. Parks JH, Worcester EM, Coe FL, et al. Clinical implications of abundant calcium phosphate in routinely analyzed kidney stones. *Kidney Int.* 2004;66:777-785.
10. Vaughan LE, Enders FT, Lieske JC, et al. Predictors of Symptomatic Kidney Stone Recurrence After the First and Subsequent Episodes. *Mayo Clin Proc.* 2019;94:202-210.
11. Gambaro G, Croppi E, Coe F, et al. Metabolic diagnosis and medical prevention of calcium nephrolithiasis and its systemic manifestations: a consensus statement. *J Nephrol.* 2016;29:715-734.
12. Curhan GC, Willett WC, Speizer FE, et al. Twenty-four–hour urine chemistries and the risk of kidney stones among women and men. *Kidney international.* 2001;59:2290-2298.
13. Worcester EM, Coe FL. Clinical practice. Calcium kidney stones. *N Engl J Med.* 2010;363:954-963.
14. Parks JH, Coward M, Coe FL. Correspondence between stone composition and urine supersaturation in nephrolithiasis. *Kidney Int.* 1997;51:894-900.
15. Ferraro PM, Ticinesi A, Meschi T, et al. Short-Term Changes in Urinary Relative Supersaturation Predict Recurrence of Kidney Stones: A Tool to Guide Preventive Measures in Urolithiasis. *J Urol.* 2018;200:1082-1087.
16. Borghi L, Meschi T, Amato F, et al. Urinary volume, water and recurrences in idiopathic calcium nephrolithiasis: a 5-year randomized prospective study. *J Urol.* 1996;155:839-843.
17. Prochaska M, Taylor E, Ferraro PM, et al. Relative Supersaturation of 24-Hour Urine and Likelihood of Kidney Stones. *J Urol.* 2018;199:1262-1266.
18. Rodriguez A, Cunha TDS, Rodgers AL, et al. Comparison of Supersaturation Outputs from Different Programs and Their Application in Testing Correspondence with Kidney Stone Composition. *J Endourol.* 2021;35:687-694.
19. Siener R, Glatz S, Nicolay C, et al. Prospective study on the efficacy of a selective treatment and risk factors for relapse in recurrent calcium oxalate stone patients. *Eur Urol.* 2003;44:467-474.
20. Borghi L, Schianchi T, Meschi T, et al. Comparison of two diets for the prevention of recurrent stones in idiopathic hypercalciuria. *N Engl J Med.* 2002;346:77-84.
21. Dhayat NA, Bonny O, Roth B, et al. Hydrochlorothiazide and Prevention of Kidney-Stone Recurrence. *N Engl J Med.* 2023;388:781-791.
22. Pottegard A, Hallas J, Olesen M, et al. Hydrochlorothiazide use is strongly associated with risk of lip cancer. *J Intern Med.* 2017;282:322-331.
23. Pedersen SA, Gaist D, Schmidt SAJ, et al. Hydrochlorothiazide use and risk of nonmelanoma skin cancer: A nationwide case-control study from Denmark. *J Am Acad Dermatol.* 2018;78:673-681 e679.

24. Haisma MS, Greven N, Logendran M, et al. Chronic Use of Hydrochlorothiazide and Risk of Skin Cancer in Caucasian Adults: A PharmLines Initiative Inception Cohort Study. *Acta Derm Venereol.* 2023;103:adv3933.

25. Borghi L, Meschi T, Guerra A, et al. Randomized prospective study of a nonthiazide diuretic, indapamide, in preventing calcium stone recurrences. *J Cardiovasc Pharmacol.* 1993;22 Suppl 6:S78-86.

26. Alonso D, Pieras E, Piza P, et al. Effects of short and long-term indapamide treatments on urinary calcium excretion in patients with calcium oxalate dihydrate urinary stone disease: a pilot study. *Scand J Urol Nephrol.* 2012;46:97-101.

27. Martins MC, Meyers AM, Whalley NA, et al. Indapamide (Natrilix): the agent of choice in the treatment of recurrent renal calculi associated with idiopathic hypercalciuria. *Br J Urol.* 1996;78:176-180.

28. Borghi L, Elia G, Trapassi MR, et al. Acute effect of indapamide on urine calcium excretion in nephrolithiasis and human essential hypertension. *Pharmacology.* 1988;36:348-355.

29. Lemieux G. Treatment of idiopathic hypercalciuria with indapamide. *Cmaj.* 1986;135:119-121.

30. Wolfgram DF, Gundu V, Astor BC, et al. Hydrochlorothiazide compared to chlorthalidone in reduction of urinary calcium in patients with kidney stones. *Urolithiasis.* 2013;41:315-322.

31. Coe FL, Parks JH, Bushinsky DA, et al. Chlorthalidone promotes mineral retention in patients with idiopathic hypercalciuria. *Kidney Int.* 1988;33:1140-1146.

32. Ettinger B, Citron JT, Livermore B, et al. Chlorthalidone reduces calcium oxalate calculous recurrence but magnesium hydroxide does not. *J Urol.* 1988;139:679-684.

33. Lockefeer JH, Juttmann JR, Birkenhager JC. The effect of long-term chlorthalidone on stone formation and stone growth, intestinal absorption of calcium and secretion of parathyroid hormone in idiopathic hypercalciuria. *Neth J Med.* 1977;20:257-262.

34. Pearle MS, Goldfarb DS, Assimos DG, et al. Medical management of kidney stones: AUA guideline. *The Journal of urology.* 2014;192:316-324.

35. Ljungberg B, Albiges L, Bedke J, et al. EAU guidelines, edn. presented at the EAU annual congress Milan 2021. *EAU Guidelines Office, Arnhem, The Netherlands. http://uroweb.org/guidelines/compilations-of-all-guidelines.* 2021.

36. Forciea MA, Starkey M. Prevention of Repeated Episodes of Kidney Stones in Adults: A Clinical Practice Guideline From the American College of Physicians.

37. Peterzan MA, Hardy R, Chaturvedi N, et al. Meta-analysis of dose-response relationships for hydrochlorothiazide, chlorthalidone, and bendroflumethiazide on blood pressure, serum potassium, and urate. *Hypertension.* 2012;59:1104-1109.

38. Ishani A, Cushman WC, Leatherman SM, et al. Chlorthalidone vs. Hydrochlorothiazide for Hypertension-Cardiovascular Events. *N Engl J Med.* 2022;387:2401-2410.

39. Roush GC, Ernst ME, Kostis JB, et al. Head-to-head comparisons of hydrochlorothiazide with indapamide and chlorthalidone: antihypertensive and metabolic effects. *Hypertension.* 2015;65:1041-1046.

40. Carter BL, Ernst ME, Cohen JD. Hydrochlorothiazide versus chlorthalidone: evidence supporting their interchangeability. *Hypertension.* 2004;43:4-9.

41. Chaffman M, Heel RC, Brogden RN, et al. Indapamide. A review of its pharmacodynamic properties and therapeutic efficacy in hypertension. *Drugs.* 1984;28:189-235.

42. Bataillard A, Schiavi P, Sassard J. Pharmacological properties of indapamide. Rationale for use in hypertension. *Clin Pharmacokinet.* 1999;37 Suppl 1:7-12.

43. Mulley BA, Parr GD, Rye RM. Pharmacokinetics of chlorthalidone. Dependence of biological half life on blood carbonic anhydrase levels. *Eur J Clin Pharmacol.* 1980;17:203-207.

44. Riess W, Dubach UC, Burckhardt D, et al. Pharmacokinetic studies with chlorthalidone (Hygroton) in man. *Eur J Clin Pharmacol.* 1977;12:375-382.

45. Harmacek D, Pruijm M, Burnier M, et al. Empagliflozin Changes Urine Supersaturation by Decreasing pH and Increasing Citrate. *J Am Soc Nephrol.* 2022;33:1073-1075.

46. Schietzel S, Bally L, Cereghetti G, et al. Impact of the SGLT2 inhibitor empagliflozin on urinary supersaturations in kidney stone formers (SWEETSTONE trial): protocol for a randomised,

double-blind, placebo-controlled cross-over trial. *BMJ Open.* 2022;12:e059073.

47. Gambaro G, Zaza G, Citterio F, et al. Living kidney donation from people at risk of nephrolithiasis, with a focus on the genetic forms. *Urolithiasis.* 2018.

48. Wason JM, Stecher L, Mander AP. Correcting for multiple-testing in multi-arm trials: is it necessary and is it done? *Trials.* 2014;15:364.

49. Pathare G, Dhayat N, Mohebbi N, et al. Acute regulated expression of pendrin in human urinary exosomes. *Pflugers Arch.* 2018;470:427-438.

50. Anderegg MA, Albano G, Hanke D, et al. The sodium/proton exchanger NHA2 regulates blood pressure through a WNK4-NCC dependent pathway in the kidney. *Kidney Int.* 2021;99:350-363.

51. Pathare G, Dhayat NA, Mohebbi N, et al. Changes in V-ATPase subunits of human urinary exosomes reflect the renal response to acute acid/alkali loading and the defects in distal renal tubular acidosis. *Kidney Int.* 2018;93:871-880.

17. APPENDICES

None.
