# Supplementary material for: Indapamide or chlorthalidone to reduce urine supersaturation for secondary prevention of kidney stones: protocol for a randomised, double-blind, cross-over trial (INDAPACHLOR)
Source: BMJ Open. 2025 Jun 16;15(6):e101594. doi: 10.1136/bmjopen-2025-101594 (PMC12314834; doi:10.1136/bmjopen-2025-101594)
Supplement: online supplemental file 2 [file bmjopen-15-6-s002.pdf]

INSELSPITAL
UNIVERSITÄTSSPITAL BERN
HÔPITAL UNIVERSITAIRE DE BERNE

University Clinic for
Nephrology and Hypertension
Director and Chief Physician
Prof. Dr. med. Bruno Vogt

Information and consent for the (additional) removal of biological material for further use in research

Dear Sir or Madam

Although biomedical research made great progress in recent decades, there are still many areas in which knowledge about the causes, detection and treatment of diseases can be improved for the benefit of the people affected. Numerous research projects in these areas can only be realized today if biological material is available. In order to be able to carry out such research projects, we would like to collect **additional biological material** from you **as part of the INDAPACHLOR study.** These samples would be taken as part of the collections already planned for the study.

The biological material :

- additional blood samples (15 to 20 ml or 1-2 tablespoons each) during 6 study appointments
- additional urine samples (20 ml each) from your 24-hour urine collection at 6 study appointments

Procedure

As the additional biological material will be taken during the sample collection already planned as part of the INDAPACHLOR study they will not be exposed to any additional health risk. However, the following inconveniences may occur during blood sampling:

- Bruising, bleeding or swelling may occur at the injection site. In rare cases, an infection may occur at the injection site.

Storage in the biobank and data protection

The biological material obtained is stored with the associated personal data in a so-called biobank and made available for research. So-called genetic data can also be obtained and stored from the samples. Genetic data can help conclusions to be drawn about your genetic make-up. The biological material and the data are stored in encrypted form. Encrypted means that all details that could identify you (e.g. name, date of birth, etc.) are replaced by a code (key) so people who do not know the code cannot draw any conclusions about you. Within the Insel Gruppe AG institution, the data can also be viewed unencrypted by authorized and clearly designated persons. The key always remains in the institution. The institution that manages the INDAPACHLOR biobank is responsible for the secure storage and protection of the biological material. The details of the biobank are set out in a set of regulations. The regulations will be provided to you as a separate document.

Your biological material and the associated personal data may only be passed on to researchers within and outside the Insel Gruppe AG institution in encrypted form. Researchers may work in Swiss or foreign institutions such as hospitals, universities or in industry.

However, the legal requirements for data protection abroad must be at least the same as in Switzerland. Future research projects with the biological material you have provided and the associated data may only be carried out with the approval of the responsible ethics committee (valid for research projects in Switzerland).

Results and incidental findings
Many research results are not relevant for the individual person. The results of research projects are usually published and can contribute to improving the treatment of future patients. Individuals will not be identifiable in a publication. However, if results are found in the research project that directly affect your health and preventive or therapeutic measures are possible, you will be informed.

Voluntariness and resignation
Your consent for the additional collection of biological material is voluntary. You can refuse the additional collection without giving reasons and without any disadvantages for any medical treatment. You can also withdraw (revoke) your consent at any time without giving reasons. In this case, your sample will be destroyed, but ongoing projects will still be completed. In such a case, please contact the institution at the address below.

Protection and loss coverage
If you suffer damage as a result of this additional removal of biological material, Insel Gruppe AG, which arranged for this additional removal and is responsible, is liable. The conditions and procedure are regulated by law. If you damage, please contact responsible person listed at the end of this document.

Take the time you need to make your decision. We will also be happy to answer your questions at any time.

consenting to the additional collection and use of biological material and the associated personal data, you are making a valuable contribution to biomedical research. If you decide to do so, we would like to thank you very much.

Name of the institution: Insel Gruppe AG

If you have any questions or suggestions, please contact (main person responsible):

Name: Prof. Dr. med. Daniel Fuster
Address: University Clinic for Nephrology, Inselspital, Bern
Phone: +41 31 632 31 44 (weekdays 8:00 - 17:00)
Email: daniel.fuster@insel.ch

Surname and first name of participant in block capitals:<br><br>Date of birth:	

Place,<br>date	Signature of participant

Surname and first name of person responsible in block capitals:	

Place,<br>date	Signature of responsible person:

This document is a translation of the original document in German called "INDAPACHLOR PIC Biobank"

This translation is endorsed by

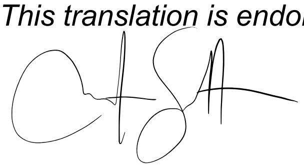

Dr. med. Martin Scoglio
Clinical and Research Fellow
University Hospital Bern
Co-investigator of the INDAPACHLOR Trial
martin.scoglio@insel.ch
