# Supplementary material for: Indapamide or chlorthalidone to reduce urine supersaturation for secondary prevention of kidney stones: protocol for a randomised, double-blind, cross-over trial (INDAPACHLOR)
Source: BMJ Open. 2025 Jun 16;15(6):e101594. doi: 10.1136/bmjopen-2025-101594 (PMC12314834; doi:10.1136/bmjopen-2025-101594)
Supplement: online supplemental file 3 [file bmjopen-15-6-s003.pdf]

University Clinic for Nephrology and Hypertension Director and Chief Physician
Prof. Dr. med. Bruno Vogt

Request to participate in medical research

INDAPACHLOR study

Study title:	Indapamide or Chlorthalidone to Reduce Urine Supersaturation for Secondary Prevention of Kidney Stones: a Randomized, Double-blind, Crossover Trial
Layman's title:	Study on the efficacy of indapamide or chlorthalidone for the reduction of urine supersaturation to prevent kidney stones

Dear Sir or Madam

We would like to inform you about the INDAPACHLOR study and ask you whether you would like to take part. Before a new drug can be prescribed by doctors, research must be carried out into how this drug works.

We call such research a **clinical study**. In this study, we want to find out what effect the study drugs indapamide and chlortalidone have on urine composition in patients with kidney stone disease.

You suffer from kidney stone disease and are already undergoing treatment for this. We would therefore like to ask you whether you would like to take part in this study.

Your participation is voluntary. The following **participant information** is intended to help you with your decision. You can ask any questions you may have about participating in the study in a **discussion with the investigator** (hereinafter referred to as the "investigator"). This is the name we give to the doctors who are responsible for a study and who will look after you as part of this study. If you wish to participate, please sign the **consent form** at the end. By signing it, you confirm that you have read and understood this information for participants. If there is anything you do not understand, please ask the investigator.

This participant information and declaration of consent consists of four parts:

Part 1 The most important facts in brief
Part 2 This is what it's all about in detail: Information on the study
Part 3 Data protection and insurance cover
Part 4 Declaration of consent

If you read **Part 1**, you will get an overview of the study. **In Part 2**, we explain the entire process and background of the study in detail. **Part 3** contains information on data and insurance protection. With your signature at the end of the document, **Part 4**, you confirm that you have understood everything and agree to participate.

In addition to this information letter, you will receive a document in we ask for your consent for additional sample collection for future research projects that have not yet been defined.

This study was initiated by Insel Gruppe AG. This institution is called the sponsor. The sponsor is responsible for, manages and finances a study.

You are responsible for this study (principal investigator):

Name: Prof. Dr. med. Daniel Fuster
Address: University Clinic for Nephrology, Inselspital Bern
Phone: +41 (0)31 632 31 44 (weekdays 8:00 - 17:00)
Email: daniel.fuster@insel.ch

Duty doctor in nephrology with 24-hour availability: +41 (0)31 664 24 06

Part 1: The most important facts in brief

1 Why are we performing this study?

Hydrochlorothiazide is used as the standard medication for kidney stone disease. This is intended to prevent the formation of kidney stones. A large Swiss study led by our clinic recently showed that hydrochlorothiazide is significantly less effective in preventing kidney stones than previously assumed.

In this study, we are investigating whether the study drugs indapamide and chlortalidone are more effective than hydrochlorothiazide for the prevention of kidney stones and whether they are well tolerated. You can find out more about the scientific background to the study in **Chapter 4**.

2. What do you have to do when you take part?

Your participation in this study will last 7 months. We will invite you for 11 study visits. Two of these appointments are part of your general treatment and take place independently of your participation in the study. The other 9 appointments are additional appointments and are only part of the study. Each appointment lasts about 15-30 minutes. After the last study visit, we will contact you once by telephone. The number of appointments is **shown in the diagram in section 5.2**.

If you decide to participate, you will be randomly assigned to one of 6 groups. All participants will receive the same 3 study drugs (indapamide, chlortalidone and hydrochlorothiazide) - but in a different order. You will not know which group you belong to.

You can find out more about the study process in **Chapter 5.**

3. What are the benefits and risks with participation?

Benefit

You will probably not benefit directly from participating in the study. However, it is possible that your participation will help future patients with kidney stones. The benefit is that statements can be made regarding the effectiveness of chlortalidone and indapamide compared to hydrochlorothiazide for the prevention of kidney stones.

Risk

The study drugs indapamide and hydrochlorothiazide are approved in Switzerland and chlorthalidone in Germany for the treatment of high blood pressure. All 3 study drugs have also been frequently used worldwide for many decades to prevent kidney stones.

You may experience side effects if you take the study drugs indapamide, chlortalidone or hydrochlorothiazide. As these drugs have been used for many decades, the risks and side effects are well known. These include:

- Decrease in the concentration of potassium in the blood
- Increase in the concentration of uric acid in the blood
- Increase in blood lipids
- allergic reactions, especially of the skin in patients with a predisposition to allergies and asthma

Chapter 6 contains further information on risks and burdens.

Part 2: This is what it's all about in detail: Information on the study

4 The scientific background of the study

4.1 Background: Why are we conducting this study?

Kidney stone disease is a common condition in younger and older people. If you develop kidney stones, you typically suffer from severe pain and may have to undergo surgical stone removal. To prevent kidney stone disease, a change in diet and a medication from the thiazide class are usually prescribed. The drugs tested in this study, hydrochlorothiazide, chlortalidone and indapamide, also belong to the thiazide class.

Thiazides are actually blood pressure-lowering drugs. However, thiazides also reduce calcium in the urine, which lowers urine supersaturation and thus inhibits the formation kidney stones. A large study in humans recently showed that the most commonly used thiazide, hydrochlorothiazide, is not very effective in preventing kidney stones. It is not known whether the drugs indapamide and chlorthalidone are more effective than hydrochlorothiazide.

In this study, we are therefore investigating whether the study drugs indapamide and chlortalidone are more effective in kidney stone disease than the study drug hydrochlorothiazide and whether they are well tolerated.

The study drugs indapamide and hydrochlorothiazide are approved in Switzerland and chlorthalidone in Germany for the treatment of blood pressure. Chlorthalidone is only approved in Switzerland as a combination product (together with another active ingredient), which is why the product from Germany (with chlorthalidone as the single active ingredient) is used for this study. Hydrochlorothiazide is also approved in Switzerland for the prevention of kidney stones. Only when the efficacy of the study drugs indapamide and chlorthalidone has been scientifically investigated and proven can they be approved and used in Switzerland for the prevention of kidney stone disease.

4.2 Structure of the study: How do we proceed?

In our study, the participants are randomly divided into groups. This is important in order to obtain reliable results from the study. This is called randomization. In each group, participants receive all 3 study drugs hydrochlorothiazide, indapamide and chlortalidone, but in a different order. In our study, there are 6 groups with 6 different sequences of study drugs. Treatment with each study drug lasts 4 weeks, with a treatment break of at least 4 weeks between each treatment phase.

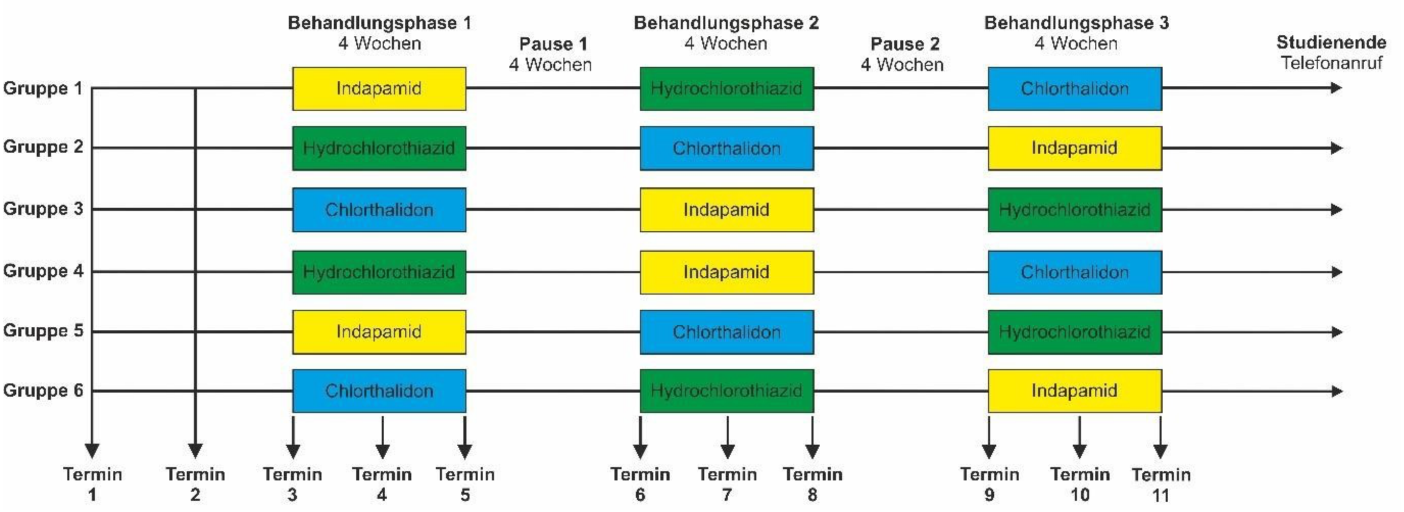

The study is a so-called double-blind study. "Double-blind" means that no one involved in conducting the study knows which group the participants have been assigned to: the participants themselves do not know which group they are in. The test persons also do not know which group individual participants belong to. In this sense, all persons involved in the study are "blind". For the study to be conducted in a blinded manner, the 3 study drugs are produced in such a way that they look the same. The idea of blinding is that everyone can have as little influence as possible on the results, whether consciously or unconsciously. Randomization and double-blinding allow us to objectively assess how well the study drugs indapamide, chlorthalidone and hydrochlorothiazide really work and whether they are safe.

43 Regulations on scientific research involving human subjects

We conduct this study in accordance with the laws in Switzerland (Human Research Act, data protection laws). We also observe all internationally recognized guidelines. The responsible ethics committee and Swissmedic have reviewed and approved the study.

Our study is a national study with a study center in Bern/Inselspital. There will be 124 participants.

A description of this study can also be found on the website of the Federal Office of Public Health at www.kofam.ch under the SNCTP registration number 000006156 or the BASEC number 2024-00477.

5. course of the study

5.1 What do you have to do if you accept to participate in the study?

Participation in the study is voluntary and lasts 7 months. You must adhere to the schedule (→ Chapter 5.2) and also to any instructions given by your investigator.

You must inform your inspector:

- if your state of health changes, e.g. if you feel worse or if you have new complaints; this also applies if you discontinue the study prematurely (→ Sections 5.3 and 5.4);
- if you are admitted to hospital or treated in hospital;
- if you want to start taking new medication;
- if your telephone number, address or e-mail address changes.

You must also note the following:

- You must effectively prevent the occurrence of pregnancy during participation (→ Chapter 5.5);
- You take the medication as explained to you by the test person;
- You will come to visits 3, 5, 6, 8, 9 and 11 (see schedule below) on an empty stomach (no food in the 6 hours before the appointment, drinking water is permitted);
- You bring all study drug vials with you to visits 5, 8 and 11.

5.2 What happens during the appointments?

During the course of your participation, you will come to us 11 times for a study visit. 2 of these appointments are part of your general treatment and will be carried out independently of your participation in the study. The other 9 appointments are additional appointments and are only part of the research study. Each appointment lasts 15-30 minutes. After the last study visit, we will contact you once by telephone. The sequence of appointments is shown in the diagram below.

We do the following for all appointments:

- We will answer your questions;
- We will ask you questions about your state of health;
- We will take about 15 ml of blood from you at a time (equivalent about 1 tablespoon).

The following also happens on individual dates (where indicated in the schedule):

- You collect your urine at home over a period of 24 hours and bring it with you to the appointment
- You give us a fresh urine sample
- We measure blood pressure and weight
- We will take an additional sample of blood (about 15-20 ml / 1-2 tablespoons) and retain a sample (20 ml) of your 24-hour urine collection for future research. You can agree to this additional blood collection and urine collection or refuse it (see the separate document "Information and consent for (additional) collection of biological material for further use in research".

These tests allow us to see how well the study drugs work and whether they are safe.

The schedule on this page shows all appointments. The **general examinations/measures are marked with a tick** (✓). The **additional examinations/measures** within the scope of the study are **marked with a plus sign** (**+**). Only these examinations are study-specific and therefore represent additional work for you.

Schedule: General and additional examinations

Study visit/ Appointment	1	2	3	4	5	6	7	8	9	10	11
Time	0	Week 2	Week 4	Week 6	Week 8	Week 12	Week 14	Week 16	Week 20	Week 22	Week 24
Duration (minutes)	30	30	30	15	30	30	15	30	30	15	30
Fasting status (f)			f		f	f		f	f		f
Examination, weight and blood pressure measurement	✓		+		+	+		+	+		+
Blood sampling		✓	+	+	+	+	+	+	+	+	+
24 hour urine collection		✓	+		+	+		+	+		+
Giving fresh urine			+		+	+		+	+		+
Pregnancy test (urine)			+			+			+		
Study visit/ Appointment	1	2	3	4	5	6	7	8	9	10	11

Time	0	Week 2	Week 4	Week 6	Week 8	Week 12	Week 14	Week 16	Week 20	Week 22	Week 24
Duration (minutes)	30	30	30	15	30	30	15	30	30	15	30
Optional: blood sampling for future research			+		+	+		+	+		+
Optional: urine collection for future research			+		+	+		+	+		+

We arrange the appointments together with you. You will receive a precise overview of the . Appointments cannot simply be postponed. We ask you to inform us quickly if you nevertheless have to postpone an appointment for important reasons.

5.3 When does participation in the study end?

Your participation will last 7 months. After the last study appointment, we will contact you again by telephone to ask you about any side effects of the study medication.

You can also cancel your participation earlier at any time (→ section 5.4). You do not have to explain why you no longer wish to participate. If you would like to end your participation earlier, please speak to your examiner.

Even if you terminate your participation prematurely, we will continue to treat and care for you medically as well as possible in accordance with current standards (→ Chapter 5.4 for alternative treatment options). In this case, we will contact you again by telephone for your safety and ask you to return all remaining study medication to us.

If you discontinue the study early, we ask you to continue to inform your investigator if your state of health changes, e.g. if you feel worse or if you have new symptoms. If your participation ends prematurely, we will still analyze the data and samples collected up to that point (e.g. blood and urine values) for the study. Your study data will remain encrypted in the database (→ Chapter 9). The blood and urine samples will be destroyed after the analysis (with the exception of any additional samples collected (→ Chapter 9.4)).

We may also have to ask you to end the study early. This could happen for various reasons:

- If your health is at risk,
- If you have to take certain medications during the study;
- In case of severe potassium deficiency;
- If you need to start therapy to lower uric acid levels;

- In the event of pregnancy;
- If the sponsor terminates the study prematurely, for example for ethical reasons, if the safety of the participants is at risk or if not enough participants can be recruited.

5.4 What happens if you don't want to take part?

Even if you do not part in this study, we will provide you with the best possible medical treatment and care in accordance with current standards. If you do not wish to take part in the study, your investigator will advise you on alternative treatment options.

5.5 Pregnancy

The study drugs can dangerous and harmful to an unborn child. For this reason, you must not have children during your participation in the study. This applies to women taking part in this study. You will discuss these issues with your investigator.

For women who can get pregnant

You must not become pregnant during your participation in the study. You must inform your partner(s) that you are taking part in this study. Before starting the study, you will take a urine pregnancy test. We will repeat the pregnancy test regularly during the study. If you are breastfeeding, you are not allowed to participate.

You must use a highly effective contraceptive method while participating in the study:

- A preparation that suppresses ovulation, either as a tablet ("pill" / "mini-pill"), syringe, stick under the skin, patch or vaginal ring
- Hormone coil
- Copper IUD, chain, ball
- Sterilization (blockage of both fallopian tubes)
- Vasectomized partner (if your partner is your only sexual partner and the vasectomy has been confirmed as successful)

If you nevertheless become pregnant during the course of the study, you must inform your investigator immediately. The investigator will then discuss the next steps with you and your pregnancy will also be monitored as part of the study.

6 Risks, burdens and side effects

6.1 What risks and burdens can occur?

There are risks and burdens associated with participation in this study, as with any medical treatment. All study drugs used are approved in Switzerland or Germany and are regularly prescribed. You will find a list of the most common risks in Chapter 6.2. Many side effects are medically treatable. We will inform you of any new findings on risks and side effects during the study.

In addition, there are risks associated with the medical examinations that we carry out in this study. You will already be familiar with some of the examinations. You will find a list of these risks in Chapter 6.3.

6.2 The most frequent and most serious risks posed by the study drugs

Here you will find information about the most common side effects that we already know about. We use the following descriptions:

very often	We find the side effect in more than 10 people out of 100 (more than 10%).
frequently	We find the side effect in 1 to 10 people out of 100 (1%-10%).

The following side effects may occur when taking indapamide: Common side effects

are

- Decrease in the concentration of potassium in the blood
- allergic reactions, especially of the skin in patients with a predisposition to allergies and asthma
- Gastrointestinal disorders (nausea, constipation)
- Dry mouth
- Headache
- Dizziness
- Tiredness
- Sensory disorders of the skin ("formication")

The following side effects may occur when taking chlorthalidone: Very common side

effects are

- Decrease in the concentration of potassium in the blood
- Increase in the concentration of uric acid in the blood
- Increase in blood lipids

Common side effects are:

- Decrease in the concentration of sodium and magnesium in the blood
- Increase in blood glucose concentration and excretion of glucose via the urine
- Deterioration of a metabolic condition
- Increase in urea and creatinine in the blood
- Headache
- Dizziness and weakness
- Decrease in blood pressure
- Palpitations
- Dizziness due to standing up too quickly
- Loss of appetite
- Dry mouth
- Gastrointestinal disorders (nausea, constipation, diarrhea, abdominal cramps, upper abdominal pain)
- Allergic skin reactions (rashes, hives, itching)
- Erectile dysfunction
- Muscle cramps and muscle weakness

The following side effects may occur when taking <u>hydrochlorothiazide</u>: Very common side effects are:

- Decrease in the concentration of potassium in the blood
- Increase in blood lipids

Common side effects are:

- Decrease in the concentration of sodium and magnesium in the blood
- Increase in the concentration of uric acid in the blood
- Decreased appetite
- Dizziness due to standing up too quickly
- Mild nausea
- Vomiting
- Hives, other skin rashes, including redness of the skin (sometimes associated with itching)
- Erectile dysfunction

6.3 Risks and burdens from examinations in the study

We carry out various medical examinations for this study (→ Chapter 5.2). These examinations are tried and tested procedures. Nevertheless, they can have risks and burdens, i.e. they can be unpleasant or have undesirable side effects. In this study there are the following risks and burdens:

- Blood collection: Bruising, bleeding or swelling may occur at the injection site. In rare cases, an infection may occur at the injection site.

7 Financing and compensation

The study is funded by the Faculty of Medicine of the University of Bern and the Inselspital. The participating researchers and the funding institutions have no direct financial benefit from conducting this study.

Participation in the study does not incur any additional costs for you or for your health insurance company. All study participants will receive CHF 200 as a lump-sum compensation for travel expenses incurred during visits to the study center.

The companies that manufacture the drugs used in the study could possibly gain a financial advantage from the results of the study in the longer term. You are not involved in this if you take part in this study.

8. Results from the study

There are results that affect you personally. Your examiner will inform you of these results. There are also incidental findings. Incidental findings are "accompanying results" that are not intended. These can unexpected blood values, for example. We will inform you if these incidental findings are relevant to your health.

There are also the overall results of the study, which come from the data of all participants. These include, for example, that we will know more about the prevention of kidney stones (→ Chapter 4.1). These results do not directly affect you or your health. However, your investigator will be happy to provide you with a summary of the overall results of the study at the end of the study if you wish.

Part 3: Data protection and insurance cover

9. Protection of data and samples

We protect your data (e.g. information such as blood pressure and pulse from your medical history) and your samples (e.g. your blood samples). There are strict legal regulations in Switzerland for the protection of data and samples.

The Swiss Data Protection Act gives you the right to information, correction and receipt of your data that collected, processed and forwarded as part of the study. These rights cannot always be guaranteed in exceptional cases due to other legal or regulatory requirements. If you have any questions, please contact your investigator.

9.1 Encryption of data and samples

Each study generates data from the examinations (e.g. blood and urine values). This data is entered into an electronic database of the sponsor. The data is encrypted. "Encrypted" means that your data cannot be linked to you via personal information such as your name, date of birth or place of residence, but only via a code. We keep a list at the hospital where it is noted which code belongs to your person. Your name, date of birth or place of residence etc. are therefore *not* directly in the database. This list remains with us at the hospital for a period of 20 years and is then destroyed. No one else receives this list. Exceptions are regulated in chapter 9.5.

All samples (e.g. blood samples) are also always encrypted in this way. Your personal data is therefore protected when we send samples to be examined in the laboratory. Even in the laboratory, the data and samples always remain encrypted.

9.2 Safe handling of data and samples during the study

The sponsor Insel Gruppe AG is responsible for the secure handling of your data and samples from this study. It is responsible for ensuring that the applicable laws, e.g. data protection laws, are complied with.

This is how the sponsor of this study protects your data:

In this study, your data will be recorded in an electronic database of the sponsor. The data is stored on a server in Switzerland and access rights are restricted to the group of people who need access to work on the study. Nevertheless, there is always a certain residual risk that third parties may access your personal data (e.g. risk of "hacking").

The blood and urine samples for this study will be analyzed on site at our hospital. From the 24-hour urine collection, 20 ml will be retained for future research if you give your separate consent. (→ Information and consent for (additional) collection of biological material for further use in research). All other residual material from the samples will be destroyed immediately after analysis.

It often important that your family doctor shares your medical history with the investigator. This also applies to other doctors who treat you. You authorize this by giving your consent at the end of the document.

9.3 Secure handling of data after the end of the study

The sponsor remains responsible for the secure handling of your data even after the end of the study. The study documents, including the data collected for the study, will be kept for at least 20 years.

Once a study has been completed, the results are usually published in scientific journals. For this purpose, the results are reviewed by other experts. Your coded data must be forwarded to these experts.

The data may not used for new research purposes. This would require your separate consent (→ Chapter 9.4).

9.4 Further use of your data in other, future studies

Your data from this study is very important for future research. The data may be used for other studies. This further use is carried out either by researchers at Inselspital or by other specialists at other institutions in Switzerland or abroad. When your data is passed on, it is always encrypted and your personal data is protected (→ Chapter 9.1).

It is possible that the data protection regulations abroad are less strict than in Switzerland. If data is transferred abroad, it is then contractually that (at least) the same data protection standards must be observed abroad as in Switzerland.

We need your separate consent for the further use of your data. This is voluntary. Please read the additional declaration of consent at the end of the document carefully.

Please sign the consent form if you would like your data to be used to support further research in the future. Even if you do not consent, you can still take part in the study. You can withdraw your decision at any time. In this case, the data will remain encrypted in the study database.

9.5 Rights of inspection during checks

The conduct of this study can be reviewed. The review is carried out by authorities such as the responsible ethics committee or the regulatory authority Swissmedic or also by foreign regulatory authorities. The sponsor must also carry out such reviews to ensure the quality of the study and the results.

A small number of specially trained people are given access to your personal data and your medical history. The data is therefore *not* encrypted for this check. The people who see your unencrypted data are subject to a duty of confidentiality.

10. Insurance cover

You are insured if you suffer damage as a result of the study - i.e. as a result of the study medication or the study procedure. The procedure is regulated by law. The sponsor has taken out insurance with Zurich Insurance Company Ltd for this purpose. If you believe that you have suffered a loss as a result of the study, please contact your investigator or the insurance company directly.

Part 4: Declarations of consent

This consent consists of two independent declarations of consent:

- Declaration of consent for participation in this study INDAPACHLOR
- Declaration of consent for the further use and disclosure of data from this study in encrypted form

Please read this form carefully. Please ask us if there is anything you do not understand or if there is anything else you would like to know. Your written consent is required for participation.

Declaration of consent for participation in the INDAPACHLOR study

**BASEC number:**	2024-00477
**Title of the study:**	Indapamide or Chlorthalidone to Reduce Urine Supersaturation for Secondary Prevention of Kidney Stones: a Randomized, Double-blind, Crossover Trial
**Layman's title:**	Study on the efficacy of indapamide or chlorthalidone for the reduction of urine supersaturation for the prevention of kidney stones
**Responsible institution:**<br>(Sponsor with address)	Insel Gruppe AG<br>Freiburgstrasse 8<br>CH-3010 Berne
**Investigation site:**	Inselspital Bern
**Principal investigator at the study site:**	Prof. Dr. med. Daniel Fuster
Surname and first name in block capitals:<br>Date of birth:	

- I received verbal and written information about the study.
- The investigator explained the purpose, procedure and risks of the study to me.
- I am taking part in the study voluntarily.
- The investigator explained to me what standard treatments are available outside of the study.
- I have had sufficient time to make this decision. I will keep the written information and receive a copy of my written declaration of consent.
- I can end my participation at any time. I do not have to explain why. Even if I end my participation, I will continue to receive my medical treatment. The data and samples that have been collected up to that point will remain stored and will be analyzed as part of the study.
- If it is better for my health, the investigator can exclude me from the study at any time.
- I understand that my data and samples will only be passed on in encrypted form. The sponsor will ensure that data protection in accordance with Swiss standards is observed.
- I will be informed of any results and/or incidental findings that directly affect my health.
- My family doctor must know that I am taking part in the study. My family doctor may share data from my medical history that is important for the study with the investigator. This also applies to other doctors who treat me.

- The responsible specialists of the sponsor, the ethics committee and / or the Swissmedic Medicines Agency may view my unencrypted data for inspection. All these persons are subject to a duty of confidentiality.
- I know that Insel Gruppe AG has taken out insurance. This insurance pays out if I suffer damage - but only if the damage is directly related to the study.

Place, date	Surname and first name of in block capitals<br><br><br>Signature of participant

Confirmation of the investigator: I hereby confirm that I have explained the nature, significance and scope of the study to this participant. I confirm that I will fulfill all obligations under Swiss law in connection with this study. Should I learn of any aspects during the course of the study that could influence the participant's willingness to take part in the study, I will inform him/her immediately.

Place, date	Surname and first name of the tester in block capitals<br><br><br>Signature of the tester

Declaration of consent to the further use and disclosure of data in encrypted form

This consent does not concern you in the sense of personal participation in a study.
"Further use" means that data can be stored beyond the time of your participation in the study and used in encrypted form for further research. This can mean, for example, that your laboratory values are statistically analyzed together with a large number of other values or that new studies are carried out on them.

"Sharing" means that your data may be passed on to other research persons or research institutions in encrypted form for further research projects. These other research persons or research institutions may also be located abroad. If the data is passed on abroad, the sponsor must ensure that (at least) the same data protection standards are observed there as in Switzerland.

**BASEC number:**	2024-00477
**Title of the study:**	Indapamide or Chlorthalidone to Reduce Urine Super-saturation for Secondary Prevention of Kidney Stones: a Randomized, Double-blind, Crossover Trial
**Layman's title:**	Study on the efficacy of indapamide or chlorthalidone for the reduction of urine supersaturation for the prevention of kidney stones
**Participant:**<br>Surname and first name in block capitals:<br>Date of birth:	

- I authorize that my coded data from this study may be used for medical research. They will then be available for future, further research projects for an indefinite period of time.
- I understand that the data is encrypted and the key is stored securely.
- The data can be analyzed in Switzerland and abroad and stored in a database here or abroad. Research institutions abroad must comply with the same data protection standards that apply in Switzerland.
- I voluntarily decide in favor of the further use and disclosure of data in encrypted form and can revoke this decision at any time. I merely inform my investigator and do not have to justify this decision. If I withdraw, the data will remain encrypted in the study database.
- Normally, all the data is analyzed together. If, by chance, there is a result that is very important for my health, I will be contacted.

Place, date	Surname and first name of in block capitals<br><br><br>Signature of participant

Confirmation of the examiner: I confirm that I have explained to the participant the nature, significance and scope of the further use and disclosure of data.

Place, date	Surname and first name of the tester in block capitals<br><br><br>Signature of the tester

This document is a translation of the original document in German called "Studieninformation & Einverständniserklärung, Version 3.0 vom 25.04.2024"

This translation is endorsed by

Dr. med. Martin Scoglio
Clinical and Research Fellow
University Hospital Bern
Co-investigator of the INDAPACHLOR Trial
martin.scoglio@insel.ch
